# Pyrrolidine, Piperazine, and Diazinane Alkaloids from the Marine Bacterium Strain *Vibrio ruber* ZXR-93

**DOI:** 10.3390/molecules29184446

**Published:** 2024-09-19

**Authors:** Xiangru Zha, Yang Li, Huange Zhao, Yinfeng Tan, Songlin Zhou

**Affiliations:** NHC Key Laboratory of Tropical Disease Control, Engineering Research Center of Tropical Medicine Innovation and Transformation of Ministry of Education, Hainan Provincial Key Laboratory of Research and Development on Tropical Herbs, School of Tropical Medicine, Hainan Medical University, Haikou 571199, China; zhaxiangru@163.com (X.Z.); liyang2308201002@163.com (Y.L.); gezi8004@163.com (H.Z.)

**Keywords:** *Vibrio ruber* ZXR-93, alkaloids derivative, structural elucidation, bioactive natural products

## Abstract

Four new alkaloids, vibripyrrolidine A (**1**), vibripiperazine A (**2**), and vibridiazinane A, B (**3**, **4**), comprising one pyrrolidine, one piperazine, and two diazinane alkaloids, along with two known analogs (**5**, **6**), were isolated from the marine bacterium *Vibrio ruber* ZXR-93 cultured in ISP2 medium. Their chemical structures were elucidated by analysis of their 1D and 2D NMR, mass spectra, and electronic circular dichroism (ECD) calculations. Compounds **1** and **3**–**6** showed vigorous antibacterial activity against *Staphylococcus aureus*, with MIC values ranging from 0.96 to 7.81 μg/mL. Moreover, compound **1** exhibited robust anti-inflammatory activity in vitro using the LPS-induced RAW264.7 macrophage model. All compounds also showed moderate antineoplastic activity against cervical cancer cells (HeLa) and gastric cancer cells (SGC-7901).

## 1. Introduction

There is an urgent need for novel and biologically active natural products to tackle the emergence of drug-resistant strains and treat various diseases that endanger human and animal health [1]. Given the constraints of land resources, we are increasingly focusing on the marine environment, which offers a more complex ecosystem and a wealth of possibilities [2]. The natural products derived from marine bacteria have been proven to possess a range of remarkable biological activities, including antibacterial [3], anti-inflammatory [4], anticancer [5], and antitrypanosomal [6] properties. These fascinating compounds are an invaluable source of novel lead drugs, offering immense potential for developing innovative therapeutic agents [7]. With their diverse and remarkable properties, marine bacteria-derived natural products are vital to unlocking the secrets of nature’s medicinal wonders [8,9,10].

**Figure 1:**
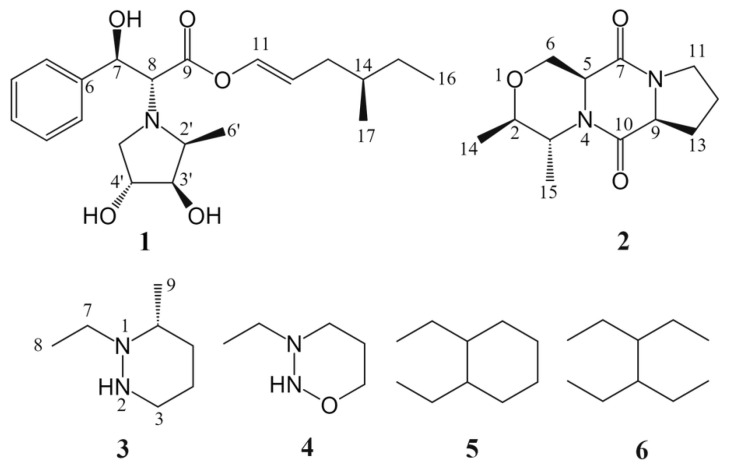
Chemical structures of compounds **1**–**6**.

Intending to discover novel natural products derived from marine bacteria, our initial step involved the isolation of cultivable marine bacteria from samples gathered from coastal habitats. Subsequently, we screened to identify strains exhibiting antibacterial and antitumor activities, prioritizing those demonstrating robust activity for further isolation and identifying their secondary metabolites. Through months of diligent research, we successfully isolated a marine bacterium, *Vibrio ruber* ZXR-93, from the coastal seawater of Haikou City, China, which exhibited significant antibacterial and antitumor activities. We then proceeded to investigate the secondary metabolites of this strain. Investigation of this bacterium cultured in ISP2 medium led to the isolation of one new pyrrolidine derivative—vibripyrrolidine A (**1**), one new piperazine derivative—vibripiperazine A (**2**), and two new diazinane derivatives—vibridiazinane A (**3**) and vibridiazinane B (**4**). Two known compounds, 1, 2-diethyl-diazinane (**5**) and tetraethyl hydrazine (**6**), are also isolated (Figure 1). All isolated metabolites (**1**–**6**) were evaluated for their antibacterial, antineoplastic, and anti-inflammatory activities in vitro, providing valuable insights into their potential therapeutic applications.

## 2. Results and Discussion

**Figure 2:**
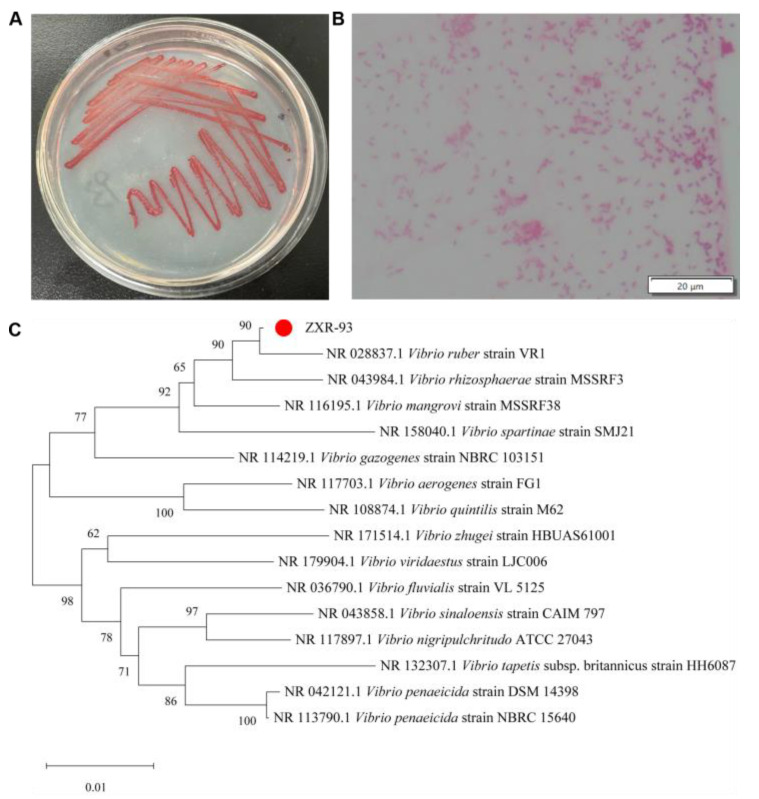
Identification of the strain ZXR-93. (**A**) colony morphology on a solid medium; (**B**) gram staining observed under a 100× light microscope; (**C**) the phylogenetic tree constructed by the strain and other bacteria.

The colonies of the ZXR-93 strain on ISP2 solid medium were red, small, and round, relatively wet, with flat edges and smooth surfaces (Figure 2A). Under the watchful eye of an optical microscope, gram staining revealed the bacterium’s morphological characteristics. It was an arc-shaped, gram-negative, produced nondiffusible, cellular red pigments (Figure 2B). The 16S rDNA sequence (1464 bp; for the detailed sequencing data, see Supporting Materials) was a perfect match with the *Vibrio ruber* sequence (KY047409.1) on GenBank, sharing a homology of 99.9% (Figure 2C). Thus, this marine bacterium was identified as *Vibrio ruber*.

**Figure 3:**
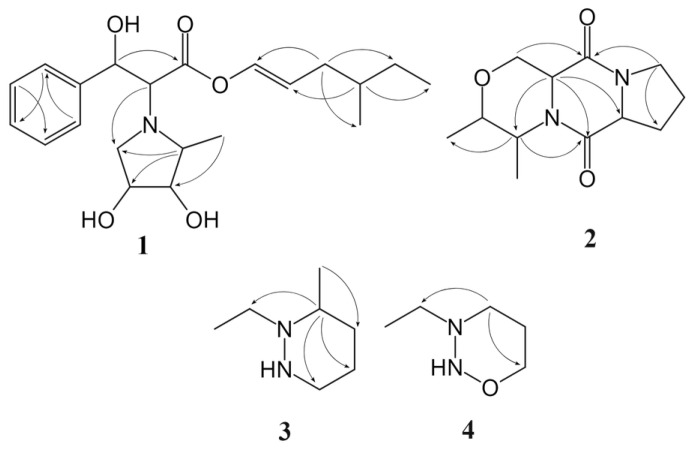
Key HMBC (black arrow) correlations of compounds **1**–**4**.

**Figure 4:**
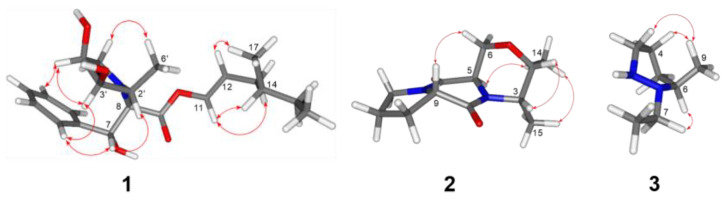
Key NOE (red arrow) correlations of compounds **1**–**3**.

**Figure 5:**
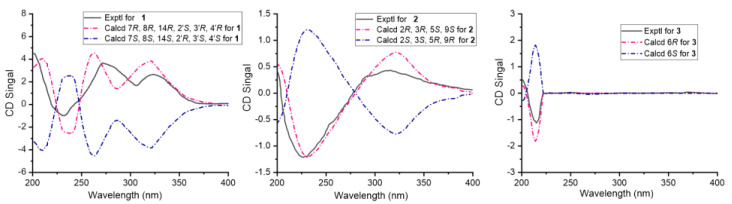
Experimental and calculational ECD spectrum of **1**–**3**.

Compound **1** was obtained as a red powder. Its molecular formula was established as C_22_H_33_NO_5_ (requiring seven degrees of unsaturation) based on high-resolution mass spectral (HR-ESIMS) analysis, which yielded a protonated molecule at *m*/*z* 392.2428 [M + H]^+^ (calcd for C_22_H_34_NO_5_, 392.2437). The ^1^H and ^13^C NMR data (Supplementary Materials Table S1) with HSQC spectrum revealed the existence of a carbonyl carbon (δC 171.7), seven doublet olefinic or aromatic signals at δH 7.29 (m), 7.07 (d, *J* = 8.5 Hz), and 6.72 (m), three oxymethines, three C–N carbons, three methynes, three methylenes, and three methyls. The planar structure of **1** was established through the analysis of HMBC spectra (Figure 3). HMBC correlations from H-8 [δH 3.65 (d, *J* = 6.9)] to C-2′ and C-5′ suggested the pyrrolidine ring was positioned at C-8. Also, HMBC correlations from H-13 (δH 3.64) to C-11, C-15, and C-17 and H-14 (δH 3.53) to C-12 and C-16 confirmed the presence of the 4-methylhex-1-ene structure. The relative configuration of **1** was determined using NOESY spectroscopy (Figure 4). Since the *J* value of H-11 is 8.5, the structure of the olefin C11-12 is *cis*, and the ROESY observed between H-11 and H-13 and H-12 and H-17 indicated that the configuration of C-14 is R-structure. NOESY correlations from protons of the benzene ring (H-2/-3/-4/-5) to H-7, H-8, H-2′, and H-3′, and from H-6′ to H-12 and H-17, suggested the relative configuration of **1** as 7*R*, 8*R*, 14*R*, 2′*S*, 3′*R*, 4′*R*. Finally, the absolute configuration **1** was determined by comparing the calculated and experimental ECD spectra. It was discovered that there was a good match between the experimental spectrum and the expected ECD spectrum of (7*R*, 8*R*, 14*R*, 2′*S*, 3′*R*, 4′*R*)-**1** (Figure 5). Thus, the absolute configuration of **1** was established with the absolute configuration of 7*R*, 8*R*, 14*R*, 2′*S*, 3′*R*, 4′*R* and named vibripyrrolidine A.

Compound **2** was isolated as a yellow powder. Its molecular formula was determined to be C_12_H_18_N_2_O_3_ based on the HR-ESIMS ion at *m*/*z* 239.1335 [M + H]^+^ (calcd for C_12_H_19_N_2_O_3_, 239.1396), indicating five degrees of unsaturation. The ^1^H and ^13^C NMR data (Table S1) with HSQC spectrum showed the presence of two carbonyl carbons (δC 171.3 and 167.7), two oxymethines [δC 69.9, δH 3.50 (m); δC 65.9, δH 4.16 (q, *J* = 6.9)], four C–N carbons, two methylenes, and two methyls. These spectroscopic features suggested that **2** belongs to the family of diketopiperazine and is very similar to Cyclo (L-Ala-L-Pro), which was usually obtained from the culture of marine-derived microorganisms and was reported to have the 5*S* and 9*S* configurations [11]. The significant differences between these two compounds were that the former added two oxymethines, one C–N carbon, and one methyl. The HMBC correlations (Figure 3) from H-2 [δH 3.50 (m)] to C-5, C-6, and C-15, from H-3 [δH 3.49 (m)] to C-5, C-6, and C-14, from H-5 [δH 4.23 (m)] to C-2 and C-3, and from H-6 [δH 4.16 (q, *J* = 6.9)] to C-2 and C-3, suggested that the six ring in **2** was formed by the connection between C-2 and C-6 via an oxygen atom. Besides, the NOESY spectrum (Figure 4) showed correlations from H-5 to H-14 and H-15, H-2 to H-3, and H-9 to H-6, which suggested that the relative configuration of **2** was 2*R*, 3*R*, 5*S*, and 9*S*. Combined with its CD spectrum (Figure 5), structure **2** was finally determined with the absolute configuration of 2*R*, 3*R*, 5*S*, and 9*S* and named vibripiperazine A.

Compound **3** was obtained as a yellow amorphous solid. Its molecular formula was determined to be C_7_H_16_N_2_ based on the HR-ESIMS ion at *m*/*z* 129.1379 [M + H]^+^ (calcd for C_7_H_17_N_2_, 129.1392), indicating one degree of unsaturation. The ^1^H and ^13^C NMR data (Table S1) with the HSQC spectrum showed the presence of two methyl groups, two methylenes, and three C-N carbons. The 1D NMR data of **3** were similar to those of a known compound that was also isolated from this strain, 1,2-diethyl-diazinane (**5**, which we named vibridiazinane C), except for the presence of an additional methyl group and the lack of a diethyl group in **3**. The HMBC correlations (Figure 3) from H-9 [δH 1.29 (d, *J* = 7.9)] to C-4, C-5, and C-7 suggested that the additional methyl group was positioned at C-6. Also, the NOESY spectrum (Figure 4) showed correlations from H-6 to H-5 and H-7 and H-9 to H-3 and H-4, which indicated that the relative configuration of **3** was 6R. Finally, its absolute configuration was determined to be 6R by the ECD calculated and experimental spectrum (Figure 5) and named vibridiazinane A.

Compound **4** was also obtained as a yellow powder. Its molecular formula was determined to be C_5_H_12_N_2_O based on the HR-ESIMS ion at *m*/*z* 117.1051 [M + H]^+^ (calcd for C_5_H_13_N_2_O, 117.1028), requiring one degree of unsaturation. The ^1^H and ^13^C NMR data suggested that structure **4** is similar to vibridiazinane A (**3**). The significant difference was the presence of an oxymethine group and the lack of a methyl group in **4**. The one-dimensional (1D) and two-dimensional (2D) NMR data analysis unambiguously accomplished detailed assignments for proton and carbon signals (Table S1). In the HMBC spectrum, correlations from H-6 to H-4 and H-7 suggested a connection between N-2 and C-4 via an oxygen atom. The structure of **4** was given the trivial name vibridiazinane B.

The structures of the known compounds 1, 2-diethyl-diazinane (**5**), and tetraethylhydrazine (**6**) were identified based on their 1H-NMR, 13C-NMR, and ESIMS data and by comparison to the reported spectroscopic data [12,13].

The antibacterial activities of the isolated compounds **1**–**6** were evaluated against four pathogenic bacteria: *Escherichia coli*, *Klebsiella pneumonia*, *Pseudomonas aeruginosa*, and *Staphylococcus aureus*. As shown in Table 1 and Figure S1, most of the compounds (**1**, **3**–**6**) had strong antibacterial effects on *S. aureus*, with MIC values ranging from 0.96 to 7.81 μg/mL, and their antibacterial effects were comparable to those of the positive control penicillin. In addition, compounds **3** and **5** had weak inhibitory effects on *K. pneumonia* and *E. coli*, but none of the compounds exhibited an antibacterial effect against *P. aeruginosa*.

The antineoplastic activities of the isolated compounds were also evaluated. The cytotoxicity of these compounds to gastric cancer cell SGC-7901, cervical cancer cell HeLa, and normal gastric mucosa cell line GES-1 was determined using an MTT assay. The results indicate that all compounds exhibit moderate antineoplastic activity against these two tumor cells but weak inhibition proliferation for normal GES-1 cells (Table 2, Figure S2). The observed cytotoxicity is concentration-dependent, with higher compound concentrations resulting in more significant inhibition of cell proliferation.

**Figure 6:**
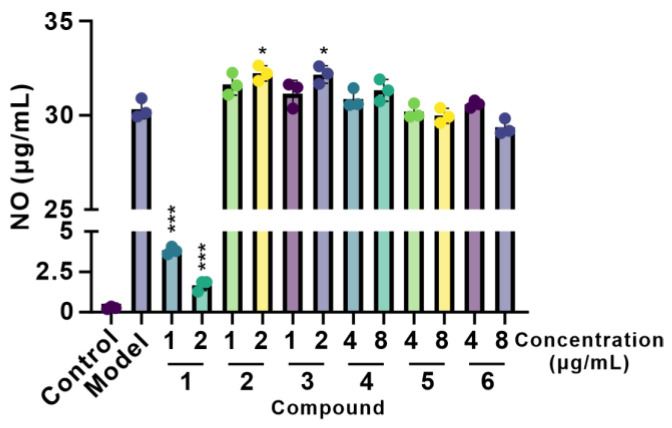
Effects of compounds on NO secretion in RAW264.7 cells. Statistical significance was determined versus the control group (one-way ANOVA followed by a Student’s *t*-test), * *p* < 0.05, *** *p* < 0.0001.

The anti-inflammatory activities of **1**–**6** were measured by accessing their influence on nitric oxide (NO) production in RAW264.7 cells induced by lipopolysaccharide (LPS). As demonstrated in Figure 6, most of the compounds (**2**–**6**) showed weak or no anti-inflammatory activity except for Vibripyrrolidine A (**1**), which revealed anti-inflammatory solid activities, with inhibition rates of 86.87% and 94.48% at concentrations of 1.0 and 2.0 µg/mL. Additionally, the cytotoxicity of these compounds on RAW264.7 cells was evaluated (Figure S3). At concentrations of compound **1**–**3** below 2 μg/mL and concentrations of compound **4**–**6** below 8 μg/mL, there was no significant difference in cell viability between the compound and the control group, indicating that these concentrations are non-toxic to cells.

## 3. Materials and Methods

### 3.1. General Experimental Procedures

ElectroSpray ionization-mass spectrometry (ESI-MS) was recorded on an AB Sciex API4000 mass spectrometer. Nuclear magnetic resonance (NMR) spectra were recorded on Bruker AV 400 MHz spectrometers with tetramethyl silane (TMS) as an internal standard. U.V. spectra were obtained on a ThermoFisher (Waltham, MA, USA) Evolution 201/220 spectrophotometer. CD spectra were recorded on a Bio-logic MOS500 circular dichroism spectrometer. Thin-layer chromatography (TLC) and precoated TLC plating were performed on SiO_2_ GF254 (10–40 mm, Qingdao Marine Chemical Inc., Qingdao, China). Column chromatography was performed on silica gel (SiO_2_: 200–300 mesh, Qingdao Marine Chemical Inc., Qingdao, China) and analytical high-performance liquid chromatography (HPLC): RP-18 (LaChrom C^18^ (4.6 × 150 mm) L-2000, Hitachi Co., Tokyo, Japan).

### 3.2. Bacterial Identification

The bacterium (strain ZXR-93) was isolated from the coastal seawater of Xinbu Island, Haikou City, Hainan Province, China (110°35′496″ E, 200°7′776″ N). Gram staining was executed using a gram staining kit (Biotechnology Co., Ltd., Shenyang, China). The staining process involved using Gentian purple for primary dyeing, iodine for mordant dyeing, a decolorization solution for decolorization, and safranin for redyeing. The morphology of bacteria was observed under a 100× oil microscope. The chromosomal DNA of the ZXR-93 strain was extracted using a bacterial genomic DNA extraction kit (Sangon Co., Shanghai, China), and the 16S rDNA fragment was amplified using a PCR kit. The PCR system consisted of 1 μL of template DNA (20–50 ng/μL), 0.5 μL of sense-27 F primer (AGTTTGATCMTGGCTCAG, 10 μM), 0.5 μL of antisense-1492 R primer (GGTTACCTTGTTACGACTT, 10 μM), 15 μL 2 × PCR Mix (Tiangen Co., Beijing, China), and double-distilled water. The PCR procedure was as follows: predenaturation at 94 °C for 5 min, denaturation at 94 °C for 45 s, renaturation at 55 °C for 45 s, extension at 72 °C for 60 s, cycle 30 times, and extension for an additional 10 min. Sangon Co. sequenced the PCR products, and the obtained 16S rDNA sequences were compared on the NCBI website. The phylogenetic tree was drawn after analysis by MEGA 11 software.

### 3.3. Fermentation, Extraction and Isolation

The bacterium was grown under revolving conditions at 28 °C, 180 rpm, for two days in 1000-mL Erlenmeyer flasks containing liquid ISP2 medium (5 g/L maltose, 4 g/L yeast powder, 4 g/L peptone, 35 g/L crude sea salt, 300 mL/flask). The fermentation broth (150 L) was divided into bacteria and supernatant by centrifugation. The supernatant was extracted with ethyl acetate 3–5 times to give an ethyl acetate solution, while the bacteria were extracted with methanol 3–5 times. Both solutions were combined and concentrated to give a crude extract (24.17 g). The crude extract was eluted on a silica gel column by gradient elution of CH_2_Cl_2_-MeOH (0–100%) to obtain 42 fractions (Fr.1-Fr.42). Fr.11 was isolated and purified twice by preparative TLC using MeOH-CH_2_Cl_2_ (1:20) and CH_2_Cl_2_-EtOAc-CHCl_3_ (3:2:1) as developing agents to yield compound **1** (20.1 mg, Rf = 0.45). Fr.18 was separated twice by preparative TLC with MeOH-CH_2_Cl_2_ (1:20) and EtOAc-CHCl_3_-nBuOH (5:5:1) to produce compound **2** (13.2 mg, Rf = 0.78). Fr.22 was subjected twice to preparative TLC by n-Hexane-CH_2_Cl_2_-MeOH-triethylamine (2:10:3:1) and acetone-triethylamine (7:1) to provide compounds **3** (19.8 mg, Rf = 0.63), **5** (47 mg, Rf = 0.21), and **6** (11.6 mg, Rf = 0.85). Compound **4** (6.7 mg, Rf = 0.34) was isolated from Fr.39 by preparative TLC using MeOH-CH_2_Cl_2_ (1:5) as a developing agent.

*Vibripyrrolidine A* (**1**): red powder, [α]D 25 + 33.7 (c 0.15, MeOH); ECD (0.025 mM, MeOH) λmax (Δε) 232 (−0.98) nm, 275 (3.57) nm, 325 (2.66) nm; U.V. (MeOH) λmax (log ε) 225 (1.60), 284 (0.66), 499 (0.79), 534 (1.29); ^1^H NMR (CD_3_OD, 400 MHz) and ^13^C NMR (CD_3_OD, 100 MHz), for data, see Table S1; HR-ESIMS *m*/*z* 392.2428 [M + H]^+^ (calcd for C_22_H_34_NO_5_, 392.2437).

*Vibripiperazine A* (**2**): yellow powder, [α]D 25 − 16.4 (c 0.15, MeOH); ECD (0.042 mM, MeOH) λmax (Δε) 227 (−1.21) nm, 315 (0.43) nm; U.V. (MeOH) λmax (log ε) 207 (2.28), 260 (0.52); ^1^H NMR (CD_3_OD, 400 MHz) and ^13^C NMR (CD_3_OD, 100 MHz), for data, see Table S1; HR-ESIMS *m*/*z* 239.1335 [M + H]^+^ (calcd for C_12_H_19_N_2_O_3_, 239.1396).

*Vibridiazinane A* (**3**): yellow crystal, [α]D 25 − 6.5 (c 0.15, MeOH); ECD (0.078 mM, MeOH) λmax (Δε) 215 (−1.12) nm; U.V. (MeOH) λmax (log ε) 215 (3.22); ^1^H NMR (CD_3_OD, 400 MHz) and ^13^C NMR (CD_3_OD, 100 MHz), for data, see Table S1; HR-ESIMS *m*/*z* 129.1379 [M + H]^+^ (calcd for C_7_H_17_N_2_, 129.1392).

*Vibridiazinane B* (**4**): yellow powder, U.V. (MeOH) λmax (log ε) 210 (2.33); ^1^H NMR (CD_3_OD, 400 MHz) and ^13^C NMR (CD_3_OD, 100 MHz), for data, see Table S1; HR-ESIMS *m*/*z* 117.1051 [M + H]^+^ (calcd for C_5_H_13_N_2_O, 117.1028).

### 3.4. ECD Calculation

The theoretical ECD spectra of compounds **1**–**3** were calculated using the Gaussian-23 software tool. Conformational analysis and density functional theory calculations were used to generate and optimize conformations at the B3LYP/6-311G level of theory, following the previously described procedure [14].

### 3.5. Antibacterial Assay

The pathogenic bacterial strains *Escherichia coli* ATCC 8739, *Klebsiella pneumonia* HMCP 805734, *Pseudomonas aeruginosa* HMCP 807623, and *Staphylococcus aureus* ATCC6538 were purchased from the National Institute for the Control of Pharmaceutical and Biological Products (Beijing, China). The specific antibacterial assay was carried out as described previously [15].

### 3.6. Cytotoxicity Assay

The human gastric cancer cell line SGC-7901, cervical cancer cell line HeLa, and the normal gastric mucosa cell line GES-1 were obtained from ATCC (Manassas, VA, USA). The cytotoxic activity against the SGC-7901, HeLa, and GES-1 cell lines was determined according to the MTT method [16].

### 3.7. Anti-Inflammatory Assay

The mouse leukemic monocyte macrophage cell line RAW 264.7 was also purchased from ATCC. The MTT assay detected the effects of these compounds on the cell viability of RAW264.7 cells. The safe concentration was selected to detect the impact of the compound on NO secretion in LPS-stimulated inflammatory cells [17].

## Figures and Tables

**Table 1:** Diameter of inhibiting bacteria circle (DIBC, mm) and MIC (μg/mL) of compounds.

	*S. aureus*		*E. coli*		*K. pneumoniae*		*P. aeruginosa*
	DIBC	MIC	DIBC	MIC	DIBC	MIC	DIBC
Compound **1**	11.8 ± 0.2	1.95	–	–	–	–	–
Compound **2**	6.9 ± 0.3	>500	–	–	–	–	–
Compound **3**	23.5 ± 0.4	0.98	7.2 ± 0.1	>500	7.8 ± 0.2	>500	–
Compound **4**	19.1 ± 0.3	3.90	–	–	–	–	–
Compound **5**	24.9 ± 0.3	0.98	7.3 ± 0.1	250	6.1 ± 0.1	250	–
Compound **6**	21.7 ± 0.3	7.81	–	–	–	–	–
Penicillin G (+)	17.8 ± 0.3	1.95	17.5 ± 0.3	3.90	25.1 ± 0.4	1.95	18.5 ± 0.3

Note: “–” indicates that no activity was detected.

**Table 2:** IC_50_ values of compounds against three cell lines (μg/mL).

	HeLa	SGC-7901	GES-1
Compound **1**	133.78 ± 17.53	279.86 ± 25.39	634.12 ± 51.35
Compound **2**	351.43 ± 77.41	1234.62 ± 89.92	1347.21 ± 97.28
Compound **3**	266.85 ± 31.60	525.25 ± 13.07	783.82 ± 49.71
Compound **4**	260.16 ± 5.68	1143.62 ± 76.29	1476.25 ± 97.82
Compound **5**	265.49 ± 16.05	387.7 ± 20.85	728.85 ± 57.86
Compound **6**	355.95 ± 21.20	266.85 ± 31.60	587.54 ± 57.86

## Data Availability

The original data presented in the study are included in the article/Supplementary Materials; further inquiries can be directed to the corresponding author.

## Supplementary Materials

The following supporting information can be downloaded at https://www.mdpi.com/article/10.3390/molecules29184446/s1: The antibacterial activity, anti-inflammatory activity, cytotoxic activity, and complete spectroscopic data for compounds **1**–**6** (PDF).
