# Supplementary material for: Pyrrolidine, Piperazine, and Diazinane Alkaloids from the Marine Bacterium Strain Vibrio ruber ZXR-93
Source: Molecules. 2024 Sep 19;29(18):4446. doi: 10.3390/molecules29184446 (PMC11433930; doi:10.3390/molecules29184446)

Supporting Information

Pyrrolidine, Piperazine, and Diazinane Alkaloids from the Marine Bacterium Strain *Vibrio ruber* ZXR-93

Xiangru Zha †, Yang Li †, Huange Zhao †, Yinfeng Tan * and Songlin Zhou *

NHC Key Laboratory of Tropical Disease Control; Engineering Research Center of Tropical Medicine Innovation and Transformation of Ministry of Education; Hainan Provincial Key Laboratory of Research and Development on Tropical Herbs, School of Tropical Medicine, Hainan Medical University, Haikou 571199, China; zhaxiangru@163.com (X.Z.);

[illegible]

* Correspondence: hy0207059@hainmc.edu.cn (Y.T.); zhousonglin106@163.com (S.Z.)

† These authors contributed equally to this work.

Contents

The 16S rDNA sequence of *Vibrio ruber* ZXR-93............S3
The physicochemical data of the known compounds **5**−**6**............S3
Table S1 ^{1}H (400 MHz) and ^{13}C (100 MHz) NMR Spectroscopic Data of **1**−**4** in CD_3OD............S4
Figure S1. Antibacterial activity of compounds **1-6** via disk diffusion method S5
Figure S2. Antibacterial activity of compounds **1-6** via two-fold dilution method............S5
Figure S3 The inhibitory effect of the compounds on two tumor cells............S6
Figure S4 The inhibitory effect of the compounds on RAW264.7 cells............S6
Figure S5 The ^{1}H NMR (400 MHz) spectrum of compound **1** in CD_3OD............S7
Figure S6 The ^{13}C NMR (100 MHz) spectrum of compound **1** in CD_3OD............S7
Figure S7 The HSQC spectrum of compound **1** in CD_3OD............S8
Figure S8 The HMBC spectrum of compound **1** in CD_3OD............S8
Figure S9 The NOESY spectrum of compound **1** in CD_3OD............S9
Figure S10 The HRESIMS spectrum of compound **1**............S9
Figure S11 The ^{1}H NMR (400 MHz) spectrum of compound **2** in CD_3OD............S10
Figure S12 The ^{13}C NMR (100 MHz) spectrum of compound **2** in CD_3OD............S10
Figure S13 The HSQC spectrum of compound **2** in CD_3OD............S11
Figure S14 The HMBC spectrum of compound **2** in CD_3OD............S11
Figure S15 The NOESY spectrum of compound **2** in CD_3OD............S12
Figure S16 The HRESIMS spectrum of compound **2**............S12
Figure S17 The ^{1}H NMR (400 MHz) spectrum of compound **3** in CD_3OD............S13
Figure S18 The ^{13}C NMR (100 MHz) spectrum of compound **3** in CD_3OD............S13
Figure S19 The HSQC spectrum of compound **3** in CD_3OD............S14
Figure S20 The HMBC spectrum of compound **3** in CD_3OD............S14
Figure S21 The NOESY spectrum of compound **3** in CD_3OD............S15
Figure S22 The HRESIMS spectrum of compound **3**............S15
Figure S23 The ^{1}H NMR (400 MHz) spectrum of compound **4** in CD_3OD............S16
Figure S24 The ^{13}C NMR (100 MHz) spectrum of compound **4** in CD_3OD............S16
Figure S25 The HSQC spectrum of compound **4** in CD_3OD............S17
Figure S26 The HMBC spectrum of compound **4** in CD_3OD............S17
Figure S27 The HRESIMS spectrum of compound **4**............S18
Figure S28 The ^{1}H NMR (400 MHz) spectrum of compound **5** in CD_3OD............S18
Figure S29 The ^{13}C NMR (100 MHz) spectrum of compound **5** in CD_3OD............S19
Figure S30 The HRESIMS spectrum of compound **5**............S19
Figure S31 The ^{1}H NMR (400 MHz) spectrum of compound **6** in CD_3OD............S20
Figure S32 The ^{13}C NMR (100 MHz) spectrum of compound **6** in CD_3OD............S20
Figure S33 The HRESIMS spectrum of compound **6**............S21

The 16S rDNA sequence of *Vibrio ruber* ZXR-93（1464 bp）:

ATTGAACGCT GGCGGCAGG CCTAACACAT GCAAGTCGAG CGGAAACGAG AGAAAGCTT

GCTTTCTCGG CGTCGAGCGG CGGACGGGTG AGTAAAGTCT GGGAAATTGC CCTGATGTGG

GGGATAACCA TTGGAAACGA TGGCTAATAC CGCATGATGT CTACGGACCA AAGAGGGGG

ACCTTCGGGC CTCTCGCGTC AGGATATGCC CAGATGGGAT TAGCTAGTTG GTGAGGTAAT

GGCTCACCAA GGCGACGATC CCTAGCTGGTC TGAGAGGATG ATCAGCCACA CTGGAACTGA

GACACGGTCC AGACTCCTAC GGGAGGCAGC AGTGGGGAAT ATTGCACAAT GGGCGCAAGC

CTGATGCAGC CATGCCGCGT GTATGAAGAA GGCCTTCGGG TTGTAAAGTA CTTTCAGCAG

TGAGGAAGGG AGTAGTTTTAA CAGAGCTGCTT TTTGACGTTA GCTGCAGAAG AAGCACCGGC

TAACTCCGTG CCAGCAGCCG CGGTAATACG GAGGGTGCGA GCGTTAATCG GAATTACTGG

GCGTAAAGCG CATGCAGGTG GTCTGTTAAG TCAGATGTGA AAGCCCGGGG CTTAACCCCG

GAGTTGCATTT GAAACTGGCA GGCTAGAGTAC TGTAGAGGGG GGTAGAATTTC AGGTGTAGCG

GTGAAATGCG TAGAGATCTG AAGGAATACC GGTGGCGAAG GCGGCCCCCT GGACAGATAC

TGACACTCAG ATGCGAAAGC GTGGGGAGCA AACAGGATTA GATACCCTGG TAGTCCACGC

CGTAAACGAT GTCTACTTGG AGGTTGTGGC CTAGAGCCGT GGCTTTCGGA GCTAACGCGT

TAAGTAGACC GCCTGGGGAG TACGGTCGCA AGATTAAAAC TCAAATGAAT TGACGGGGGC

CCGCACAAGC GGTGGAGCAT GTGGTTTAATT CGATGCAACG CGAAGAACCT TACCTACTCTT

GACATCCAGA GAAGCCGGA AGAGATTCTG GTGTGCCTTC GGGAGCTCTG AGACAGGTGC

TGCATGGCTG TCGTCAGCTC GTGTTGTGAA ATGTTGGGTT AAGTCCCGCA ACGAGCGCAA

CCCTTATCCTT GATTGCCAGC ACTTCGGGTG GGAACTTCAG GGAGACTGCC GGTGATAAAC

CGGAGGAAGG TGGGGACGAC GTCAAGTCATC ATGGCCCTTAC GAGTAGGGCT ACACACGTGC

TACAATGGCG TATACAGAGG GCAGCTAACT TGCGAGAGTG TGCGAATCCC AAAAAGTAC

GTCGTAGTCC GGATTGGAGT CTGCAACTCG ACTCCATGAA GTCGGAATCG CTAGTAATCG

TAGATCAGAA TGCTACGGTG AATACGTTCC CGGGCCTTGT ACACACCGCC CGTCACACCA

TGGGAGTGGG CTGCAAAAGA AGCAGGTAGT TTAACCTTCG GGAGGACGCT TGCCACTTTG

TGGTTCATGAC TGGGGTG

Compound **5**: yellow powder, HRESIMS *m/z* 143.2460 [M+H]+) (calculated for $C_8H_{19}N_2$, 143.2499), ^{1}H NMR (400 MHz, CD_3OD) δ 3.16, (2H, t, H-3), 1.35, (2H, t, H-4), 1.35, (2H, t, H-5), 3.16, (2H, t, H-6), 3.21 (2H, q, H-7), 1.26 (3H, t, H-8), 3.21 (2H, q, H-9), 1.26 (3H, t, H-10); ^{13}C NMR (100 MHz,

CD_3OD) δ 52.3, (C-3), 29.4 (C-4), 29.4 (C-5), 52.3, (C-6), 46.5 (C-7), 7.9 (C-8), 46.5 (C-9), 7.9 (C-10).

Compound **6**: yellow powder, HRESIMS *m/z* 145.2620 [M+H]+) (calculated for $C_8H_{21}N_2$, 145.2657), ^{1}H NMR (400 MHz, CD_3OD) δ 3.19, (2H, q, H-3), 1.29, (3H, t, H-4), 3.19, (2H, q, H-5), 1.29, (3H, t, H-6), 3.19 (2H, q, H-7), 1.29 (3H, t, H-8), 3.19 (2H, q, H-9), 1.29 (3H, t, H-10); ^{13}C NMR (100 MHz, CD_3OD) δ 46.5, (C-3), 7.9 (C-4), 46.5 (C-5), 7.9, (C-6), 46.5 (C-7), 7.9 (C-8), 46.5 (C-9), 7.9 (C-10).

TABLE

Table S1 ^{1}H (400 MHz) and ^{13}C (100 MHz) NMR Spectroscopic Data of **1**−**4** in CD_3OD

No.	Compound **1**		Compound **2**		Compound **3**		Compound **4**	
	δ H (*J* in Hz)	δC type	δ H (*J* in Hz)	δC type	δ H (*J* in Hz)	δC type	δ H (*J* in Hz)	δC type
1	7.29,m	126.7, CH						
2	7.29, m	129.0, CH	3.50, m	69.9, CH				
3	7.29, m	128.2, CH	3.49, m	50.7, CH	3,21, m	52.3, CH_2		
4	7.29, m	129.0, CH			1.34, m	29.4, CH_2	3.82, m	69.9, CH_2
5	7.29, m	126.7, CH	4.23, t (8.1)	53.1, CH	1.31, m	30.5, CH_2	1.31, m	29.4, CH_2
6		134.4, C	4.16, q (6.9)	65.9, CH	3.46, m	62.3, CH	3.61, m;	65.9, CH_2
7	3.73, m	69.8, CH		171.3, C	3,17, m	46.5, CH_2	3.28, m	47.7, CH_2
8	3.65, d (6.9)	65.9, CH			1.26, m	6.4, CH_3	1.16, m	13.8, CH_3
9		171.7, C	4.23, t (8.1)	59.2, CH	1.29, d	7.9, CH_3		
10				167.7, C				
11	7.07, d (8.5)	129.9, CH	3.47, m	45.1, CH_2				
12	6.72, m	114.9, CH	1.98, m	22.3, CH_2				
13	3.64, m	40.9, CH_2	2.27, m	27.8, CH_2				
14	3.53, m	39.5, CH	1.34, d (0.8)	14.4, CH_3				
15	1.29, m	29.4, CH_2	1.36, d (0.8)	13.9, CH_3				
16	0.90, m	13.1, CH_3						
17	1.09, d (6.5)	17.2, CH_3						
2′	3.56, m	51.0, CH						
3'	4.75, m	74.7, CH						
4'	3.66, m	68.8, CH						
5'	3.35, d (1.4)	48.5, CH_2						
6'	1.17, d (6.4)	13.9, CH_3						

FIGURES

Figure S1. Antibacterial activity of compounds **1-6** via disk diffusion method. (A) *S. aureus*; (B) *E. coli*; (C) *K. pneumoniae*; (D) *P. aeruginosa*. "+" is penicillin G, and "－" is chromatographic-grade methanol.

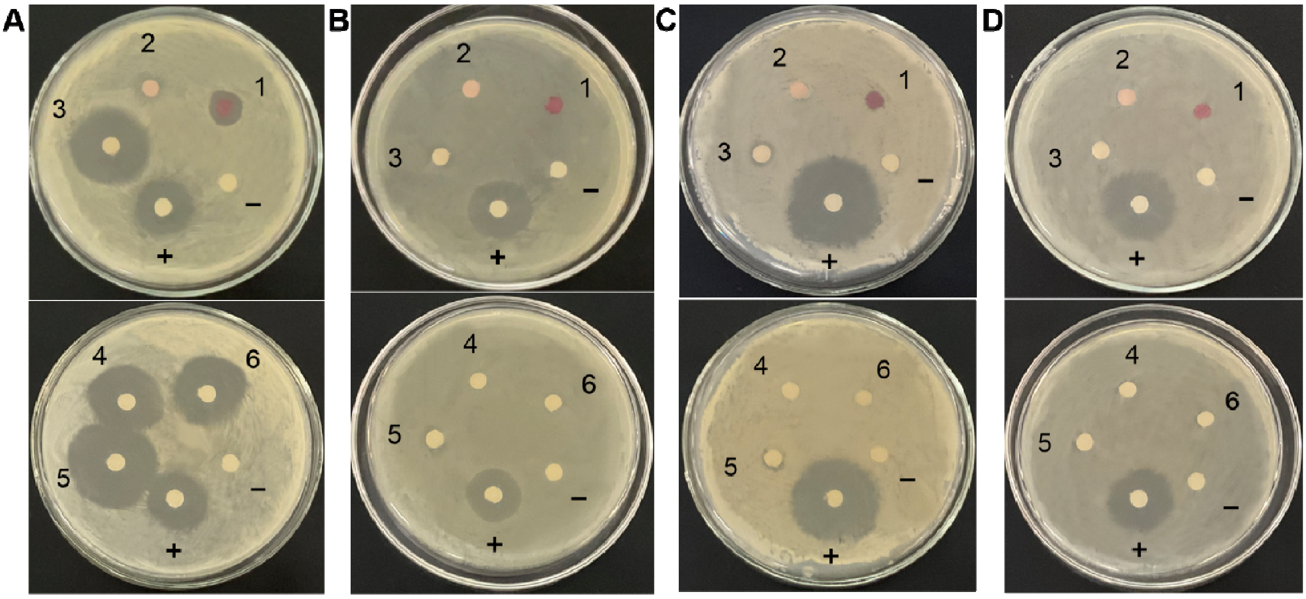

Figure S2. Antibacterial activity of compounds **1-6** *via* two-fold dilution method. (A) *S. aureus*; (B) *E. coli*; (C) *K. pneumonia*.

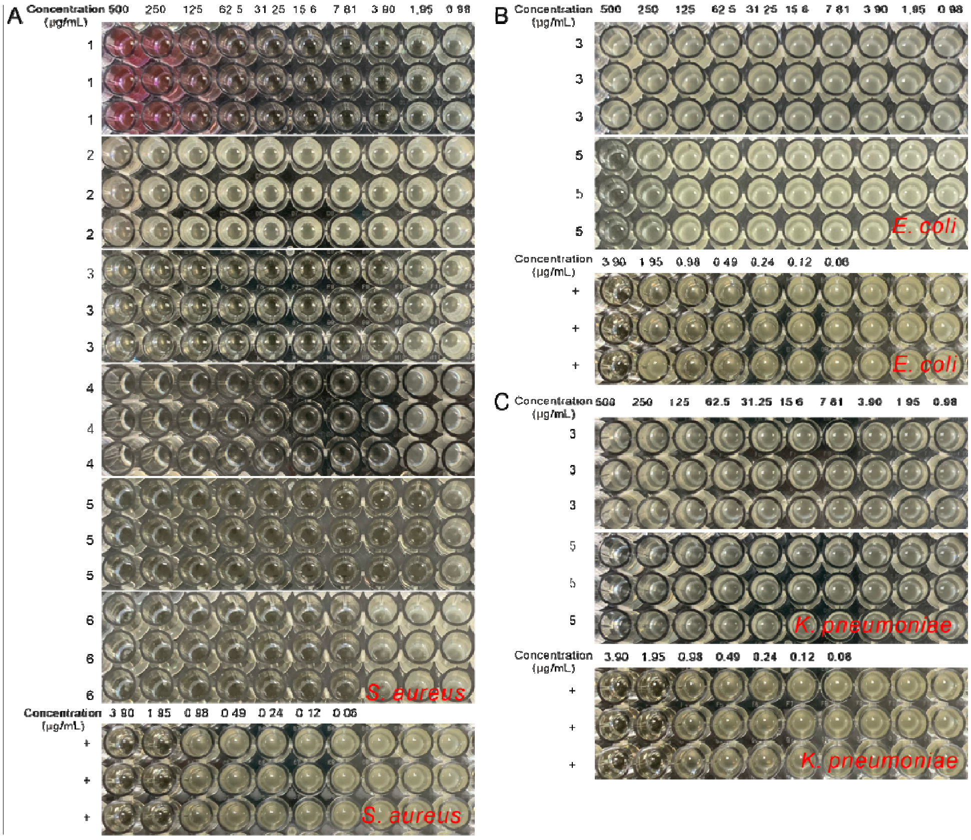

Figure S3. The inhibitory effect of the compounds **1-6** on two tumor cells. (A) Compound **1**, (B) Compound **2**, (C) Compound **3**, (D) Compound **4**, (E) Compound **5**, (F) Compound **6**.

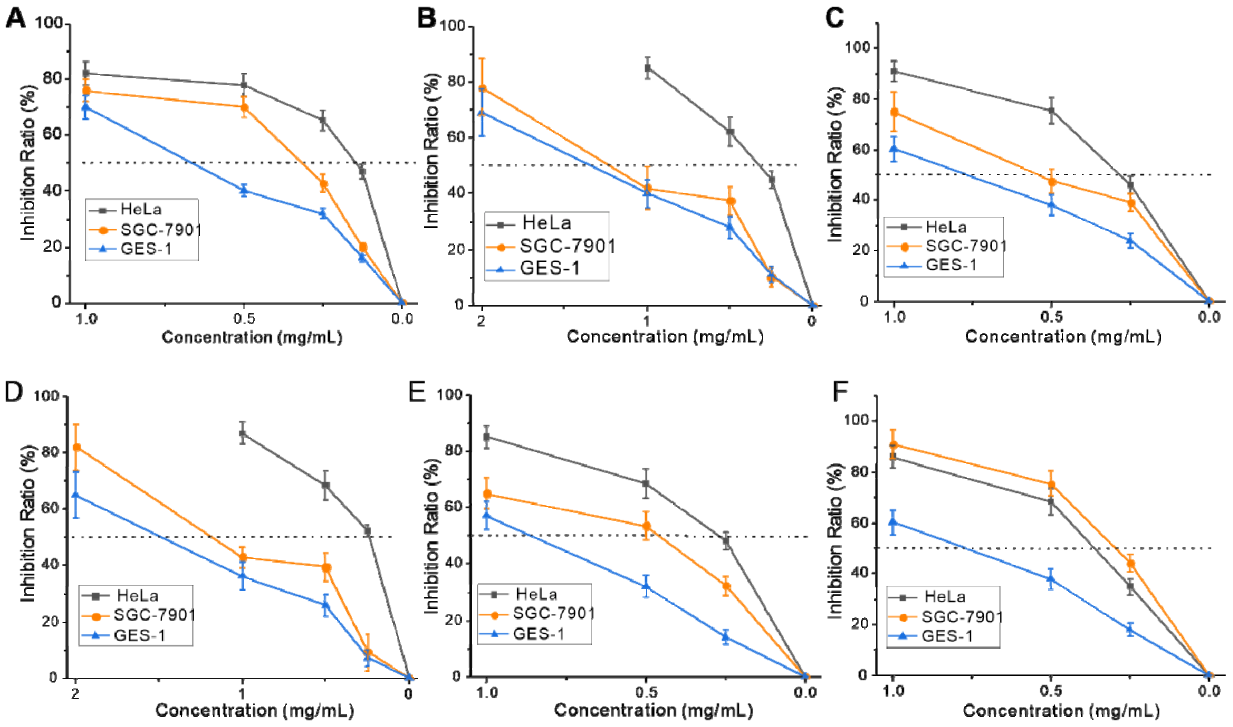

Figure S4. The inhibitory effect of the compounds **1-6** on RAW264.7 cells. (A) Compound **1**, (B) Compound **2**, (C) Compound **3**, (D) Compound **4**, (E) Compound **5**, (F) Compound **6**. Statistical significance was determined versus the control group (one-way ANOVA followed by a Student's t test): *P < 0.05, **P < 0.01, ***P < 0.001.

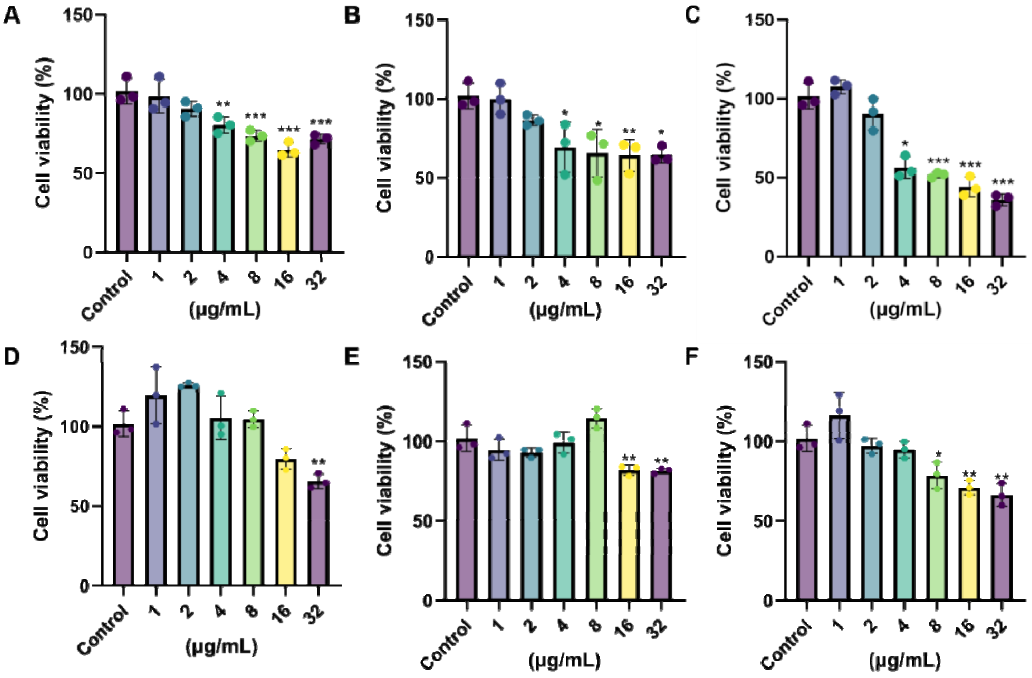

Figure S5. The ^{1}H NMR (400 MHz) spectrum of compound **1** in CD_3OD

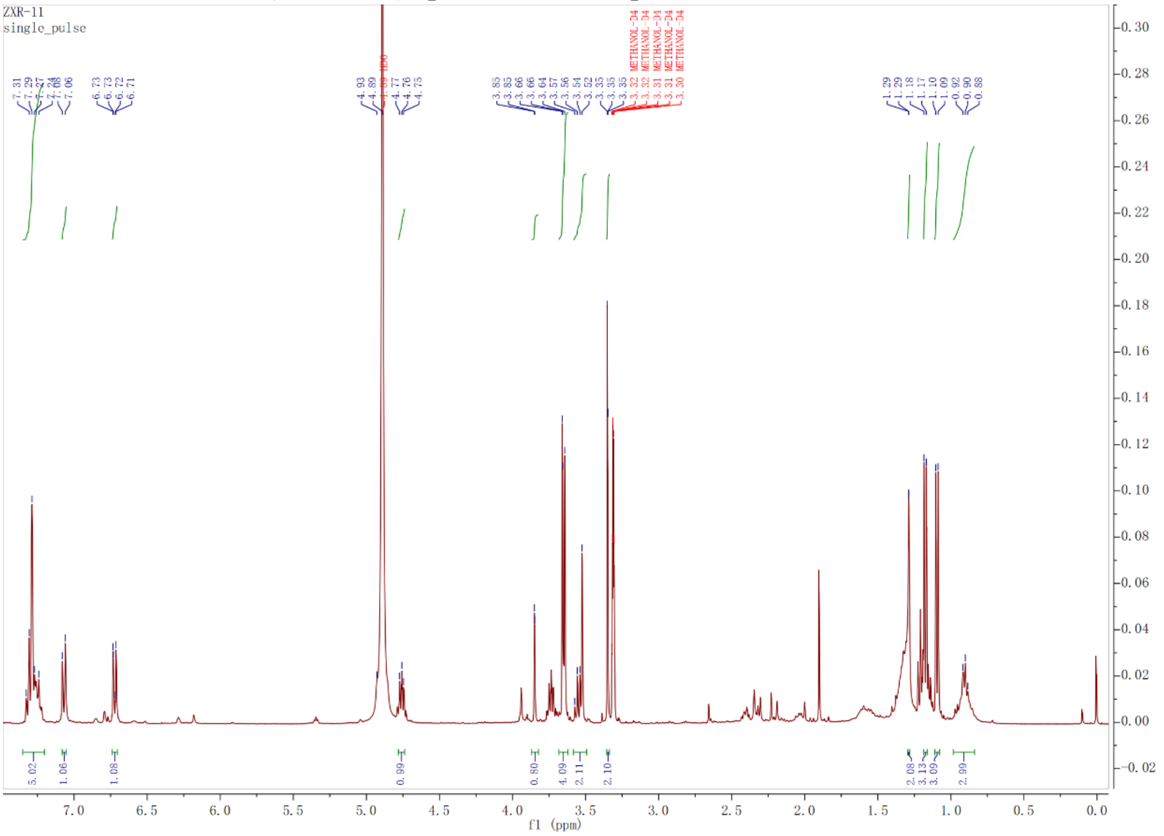

Figure S6. ^{13}C NMR (100 MHz) spectrum of compound **1** in CD_3OD

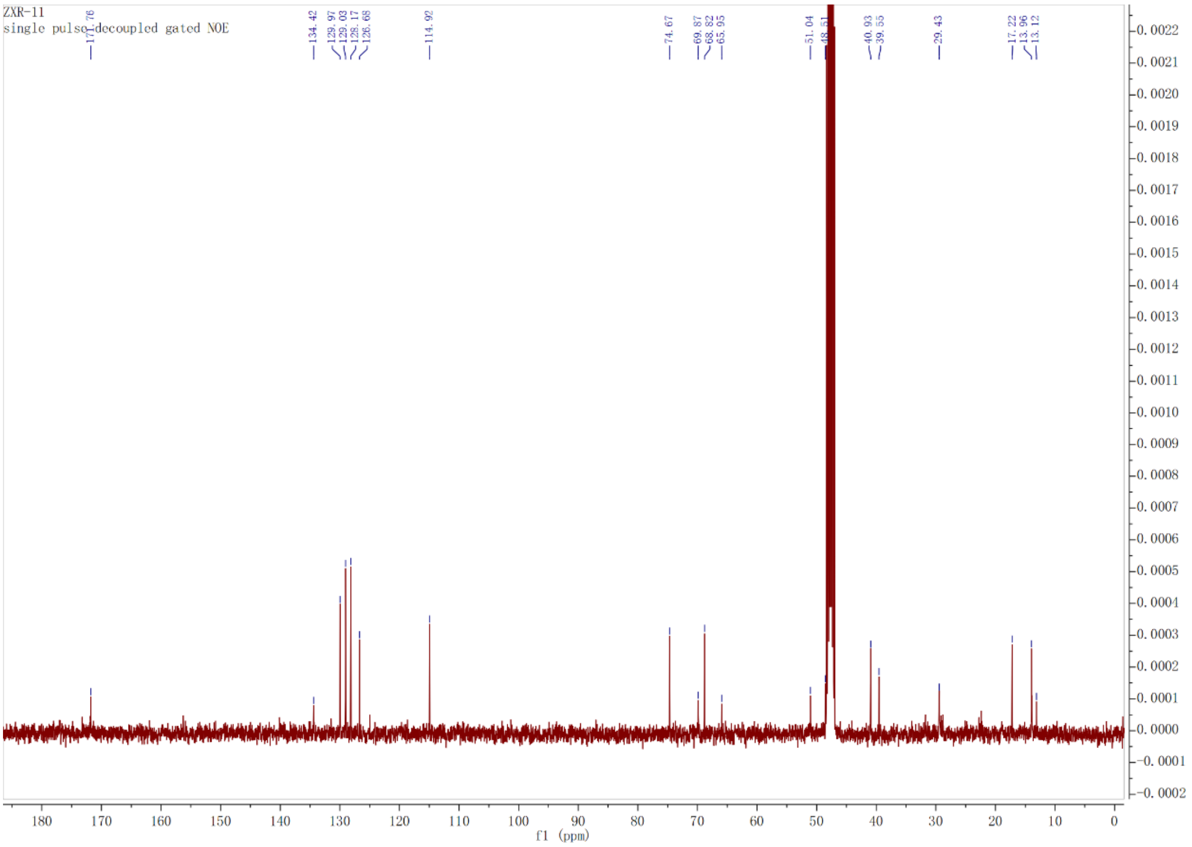

Figure S7. The HSQC spectrum of compound **1** in CD_3OD

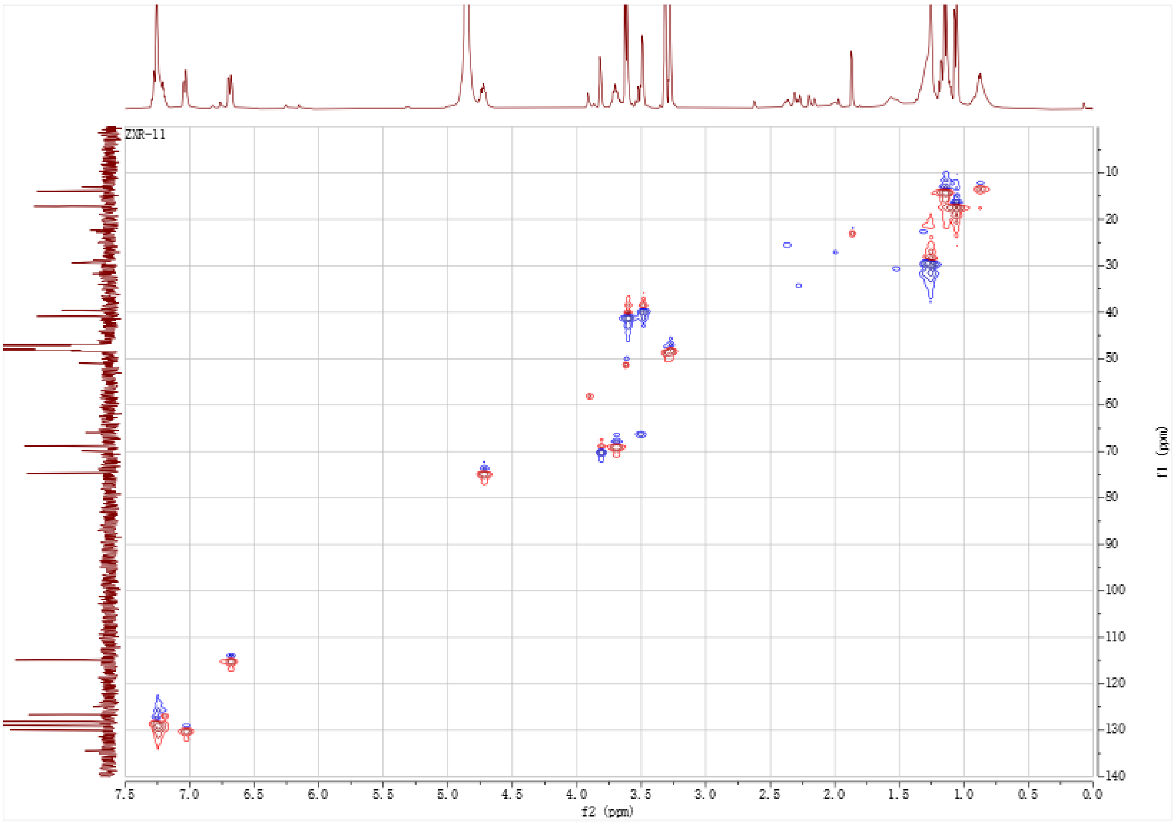

Figure S8. The HMBC spectrum of compound **1** in CD_3OD

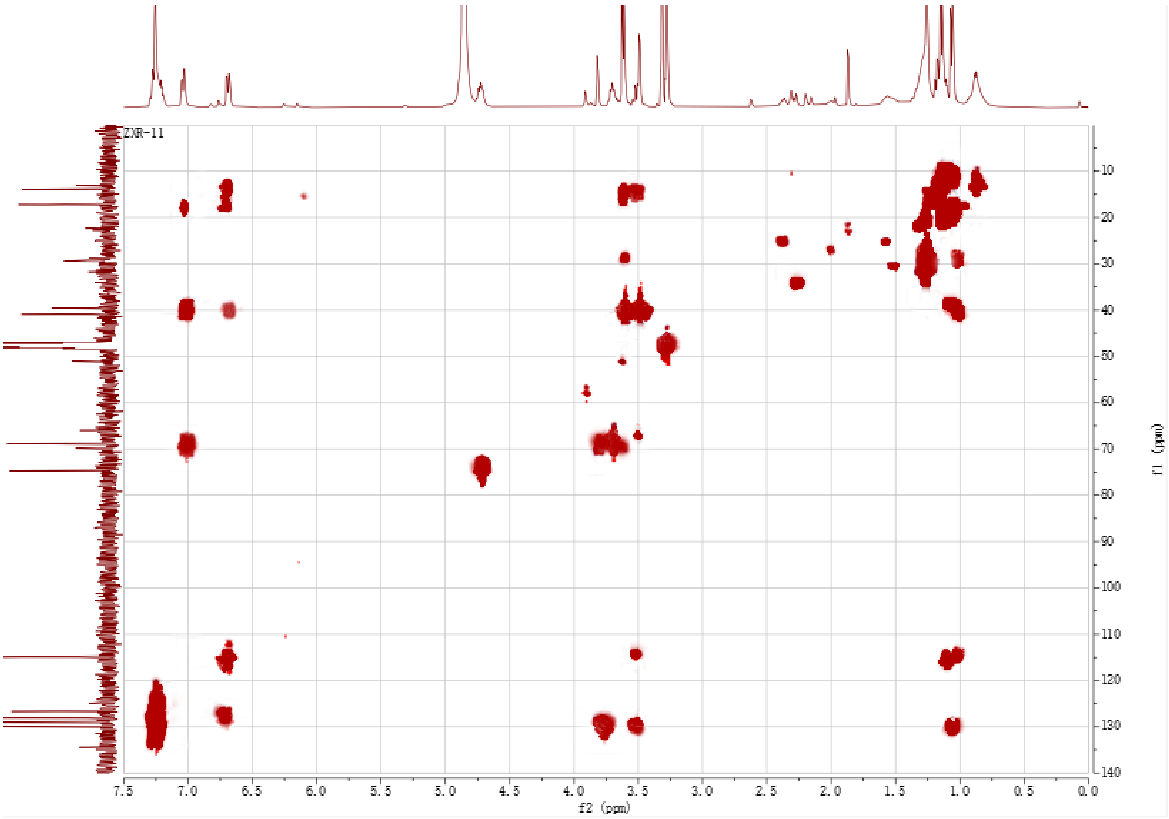

Figure S9. The NOESY spectrum of compound **1** in CD_3OD

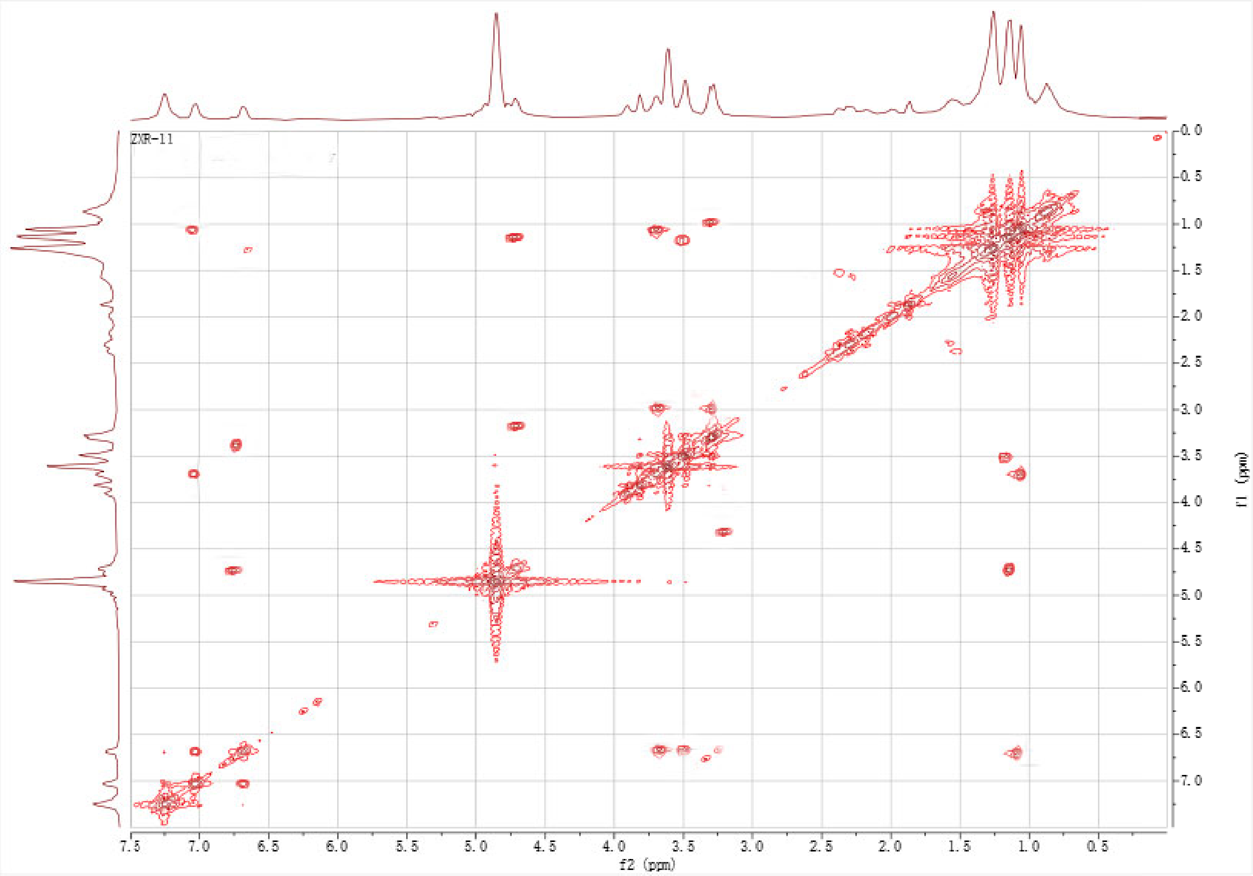

Figure S10. HRESIMS spectrum of compound 1

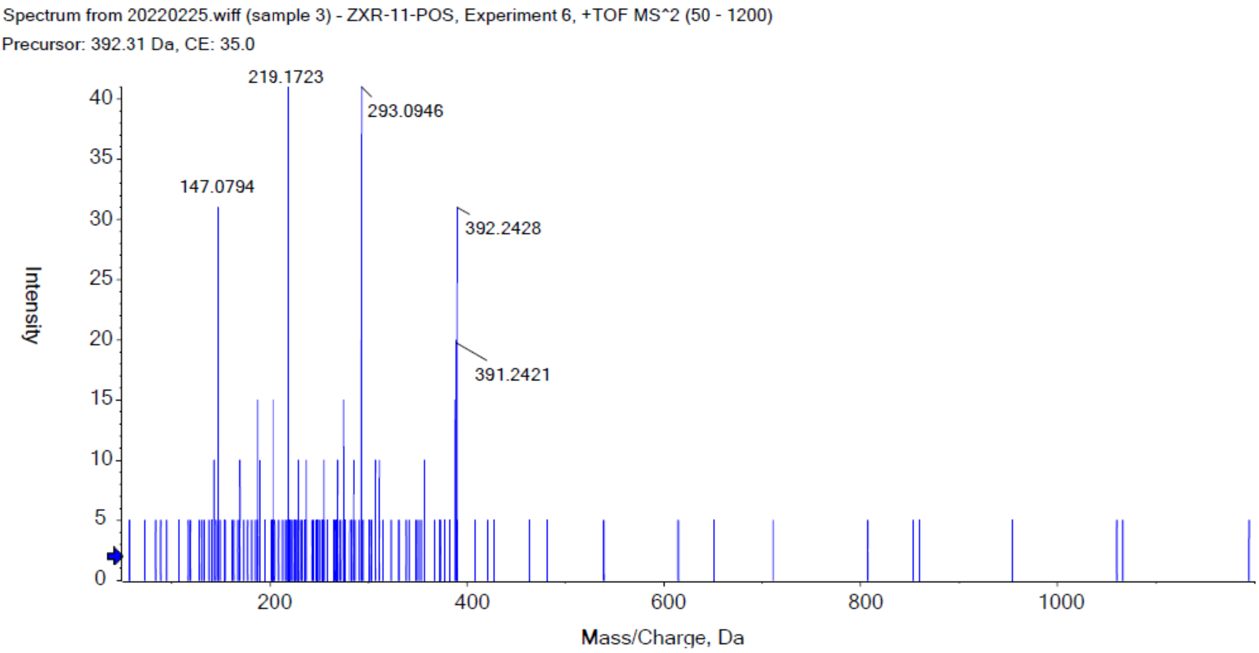

Figure S11. The ^{1}H NMR (400 MHz) spectrum of compound **2** in CD_3OD

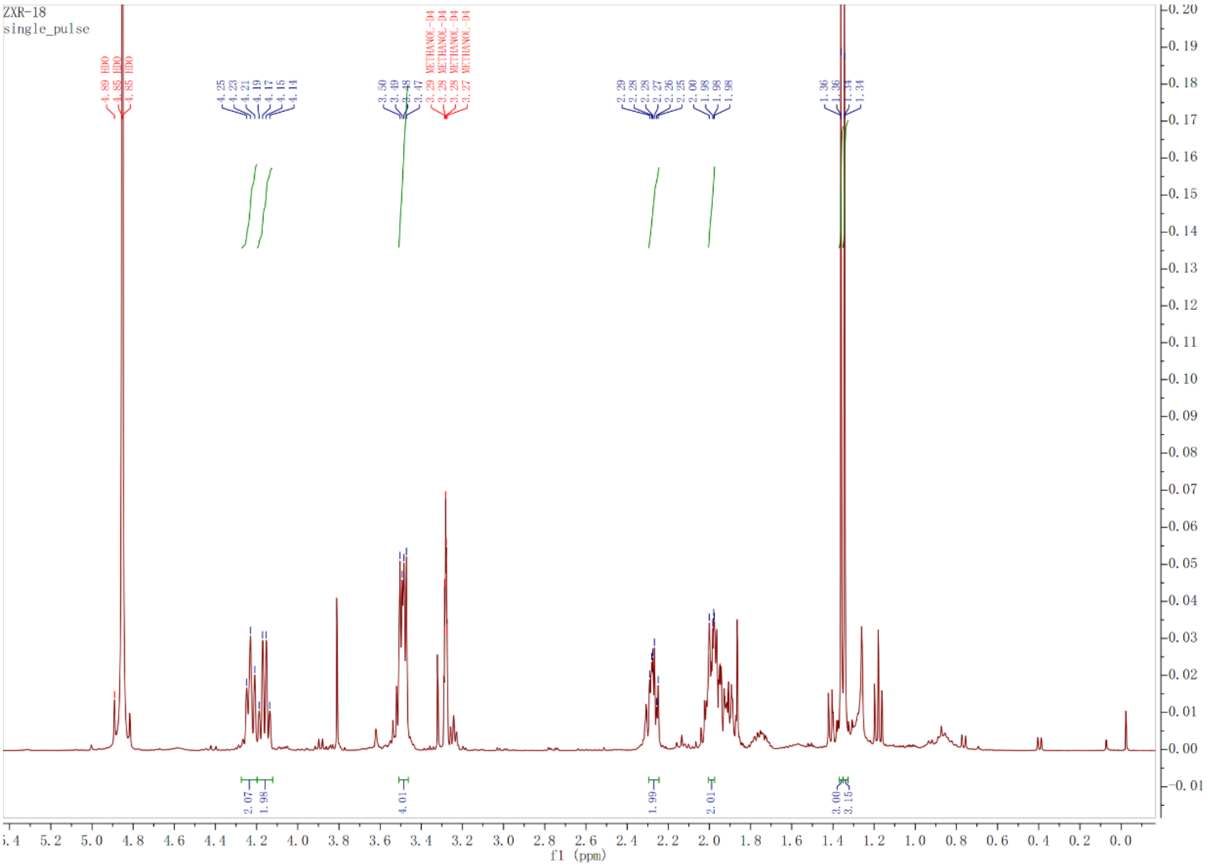

Figure S12.The ^{13}C NMR (100 MHz) spectrum of compound **2** in CD_3OD

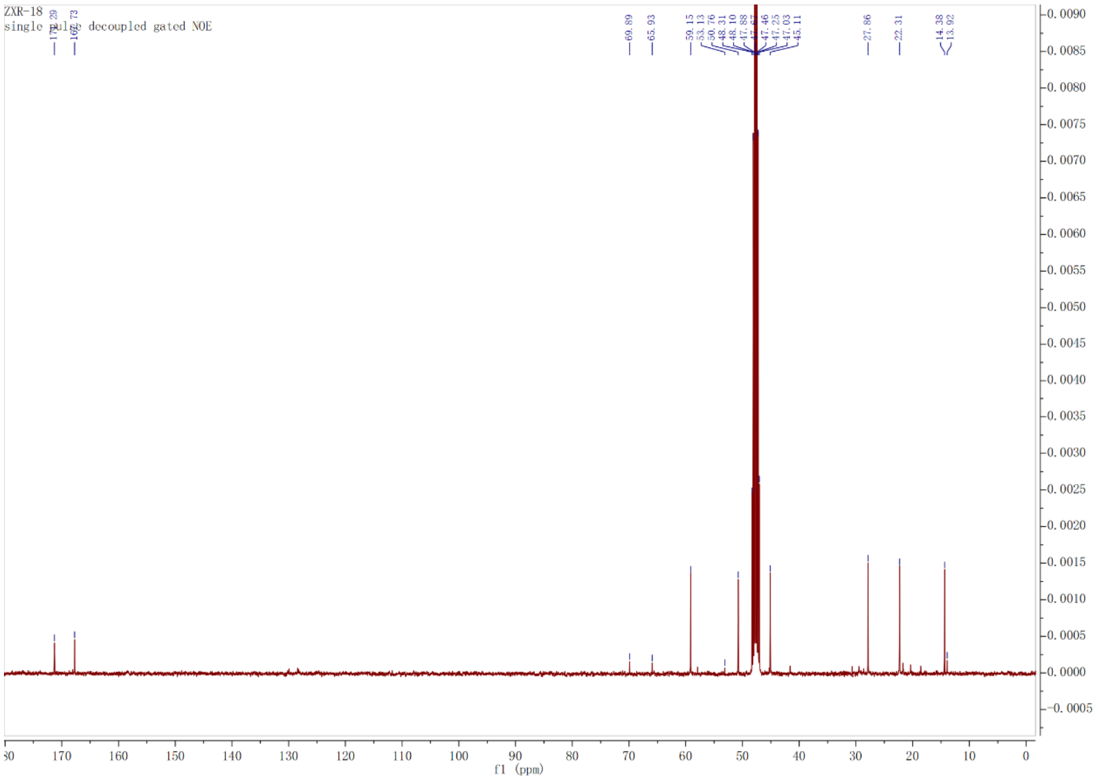

Figure S13. The HSQC spectrum of compound **2** in CD_3OD

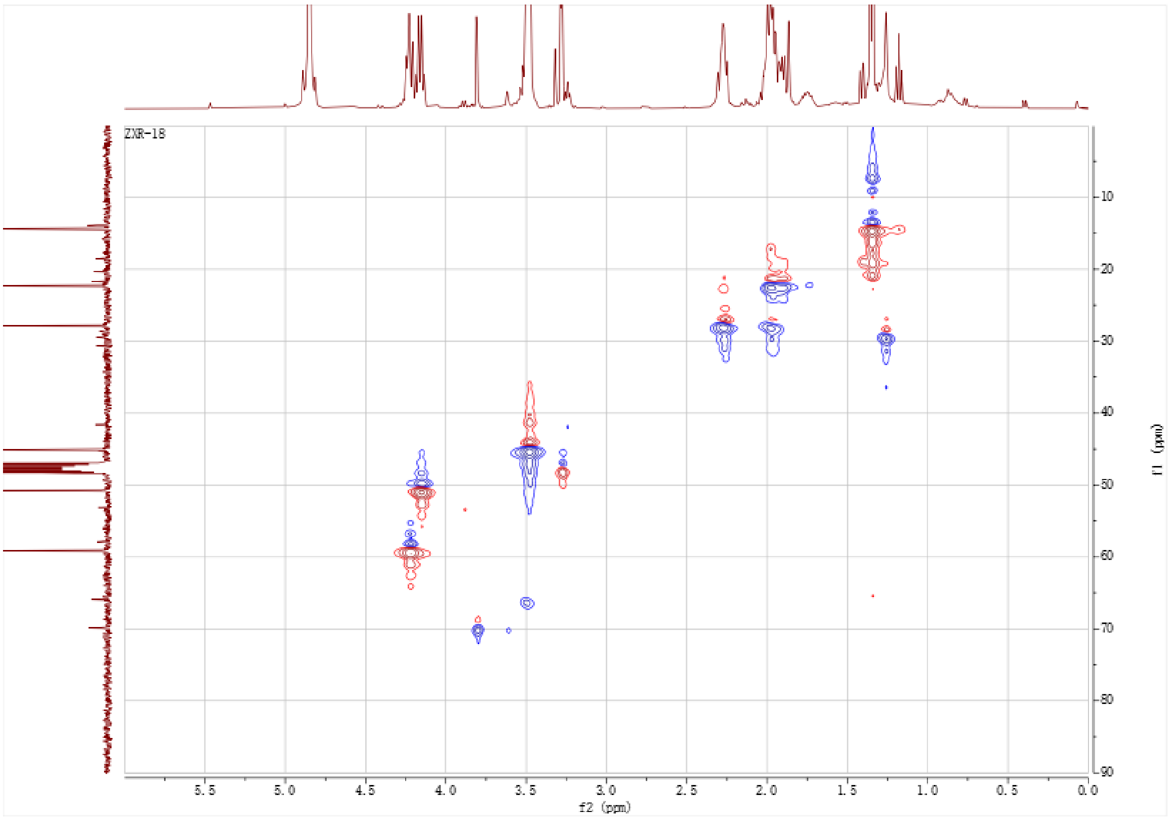

Figure S14. The HMBC spectrum of compound **2** in CD_3OD

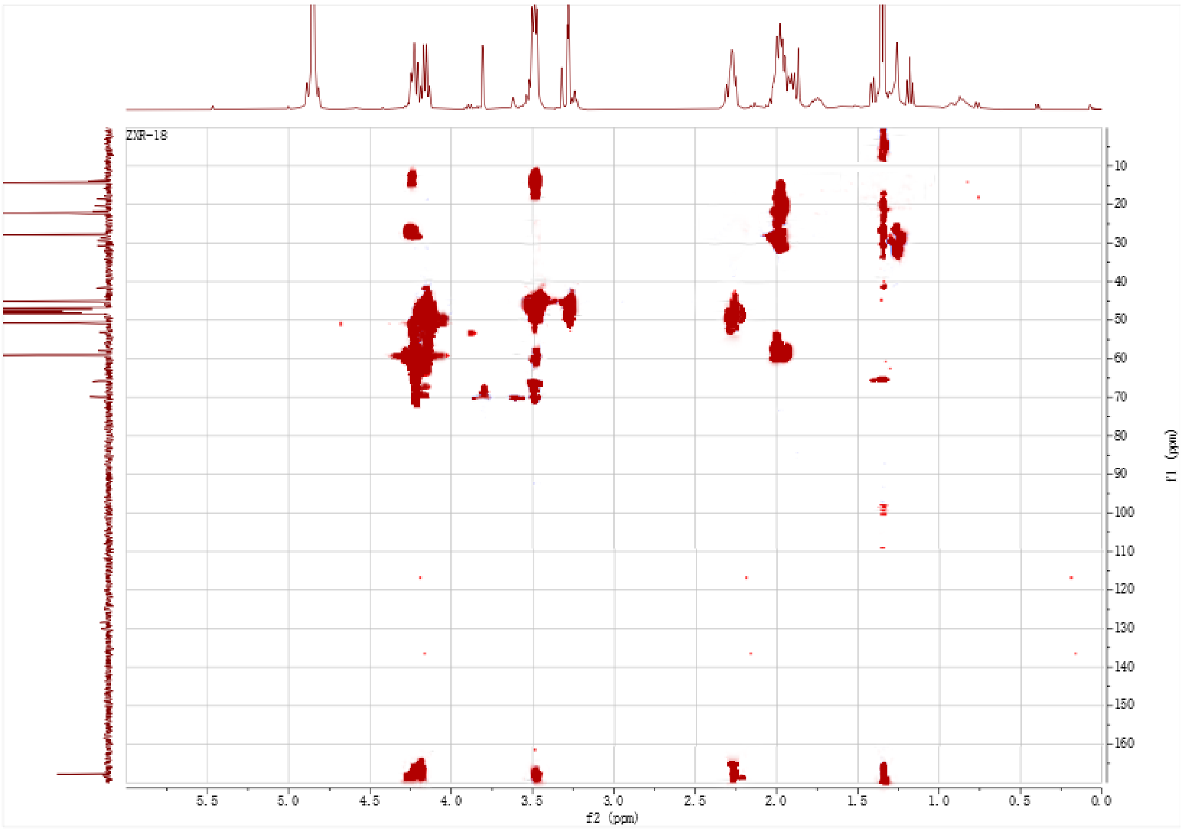

Figure S15. The NOESY spectrum of compound **2** in CD_3OD

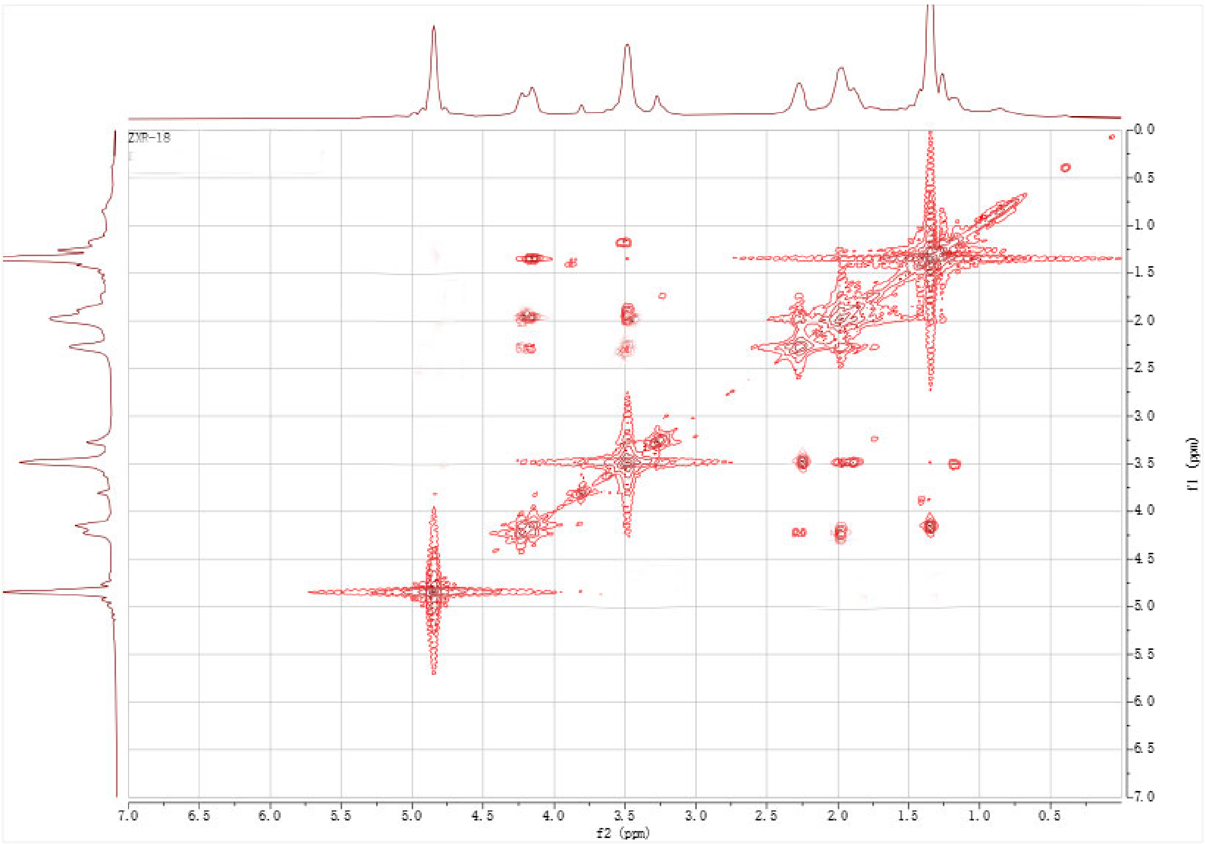

Figure 16. The HRESIMS spectrum of compound **2**

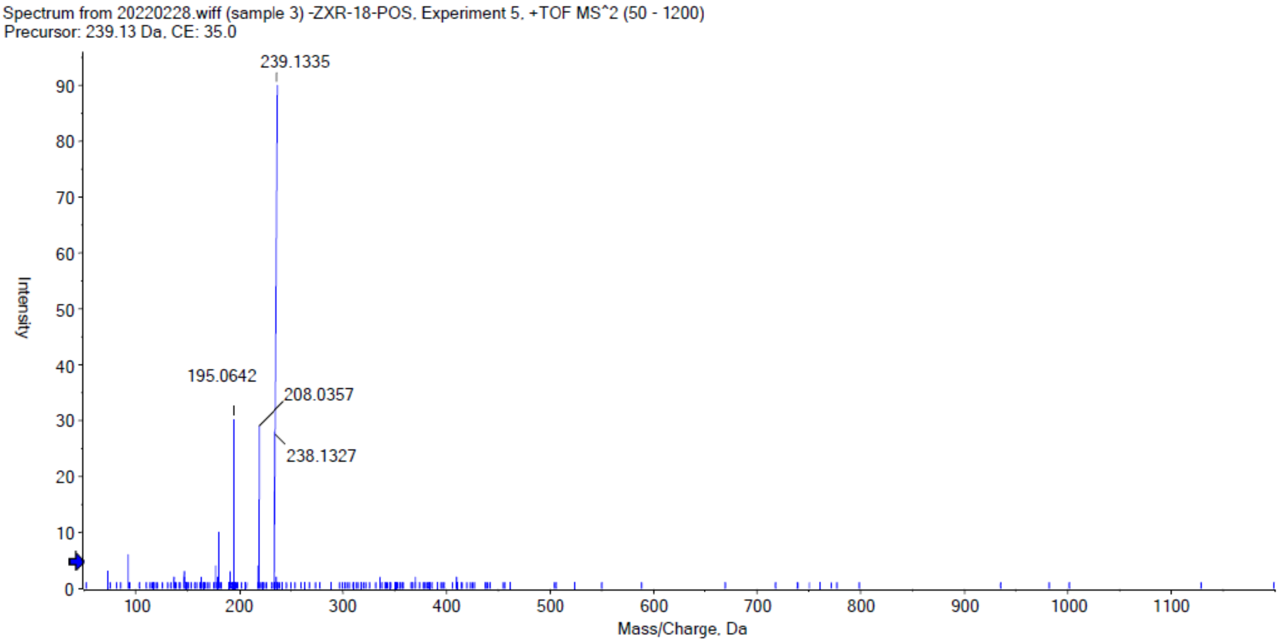

Figure S17. The ^{1}H NMR (400 MHz) spectrum of compound **3** in CD_3OD

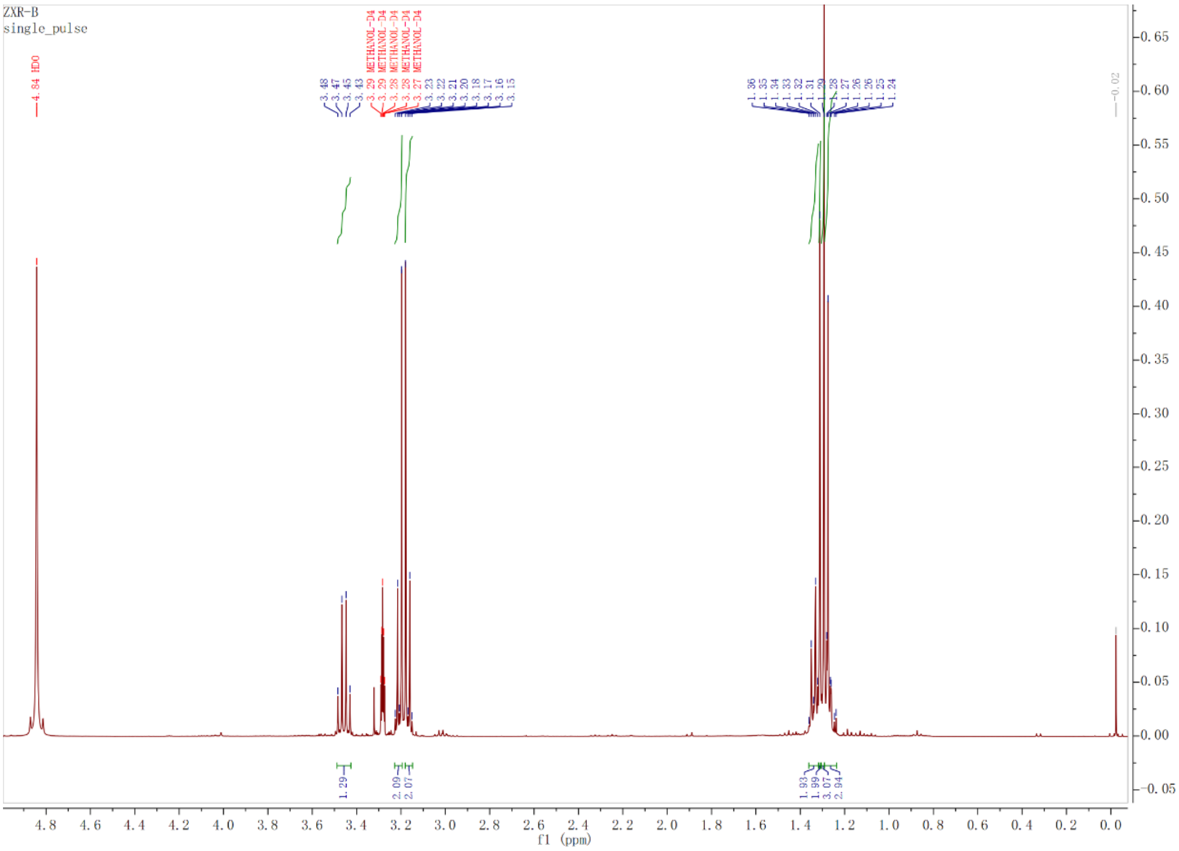

Figure S18. The ^{13}C NMR (100 MHz) spectrum of compound **3** in CD_3OD

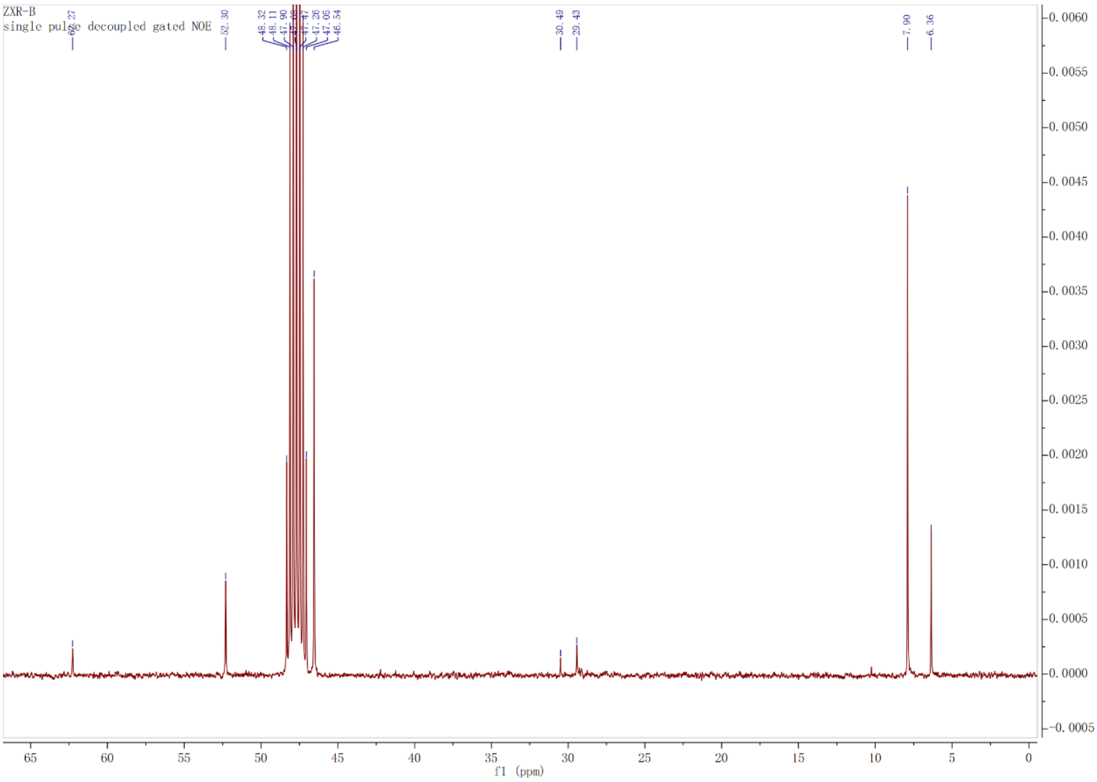

Figure S19. The HSQC spectrum of compound **3** in CD_3OD

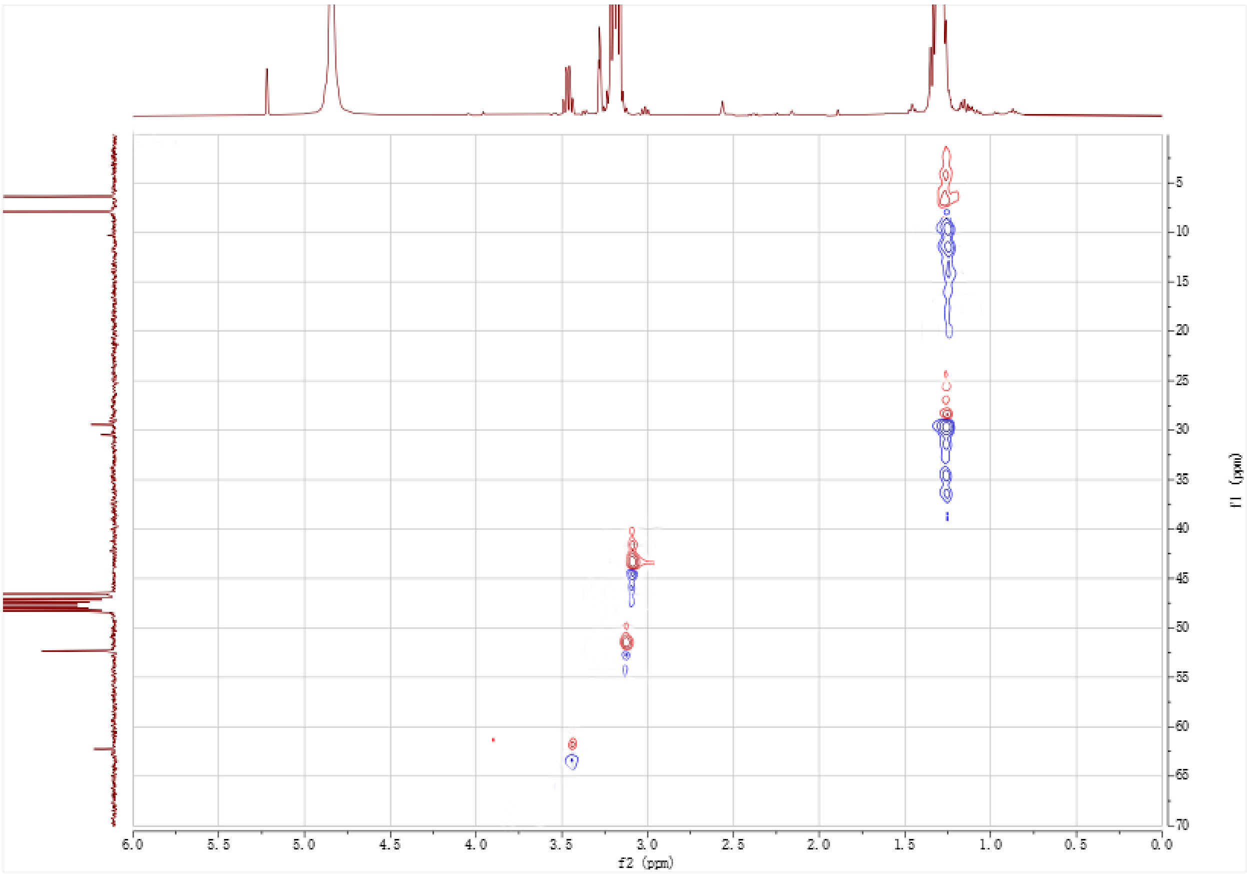

Figure S20. The HMBC spectrum of compound **3** in CD_3OD

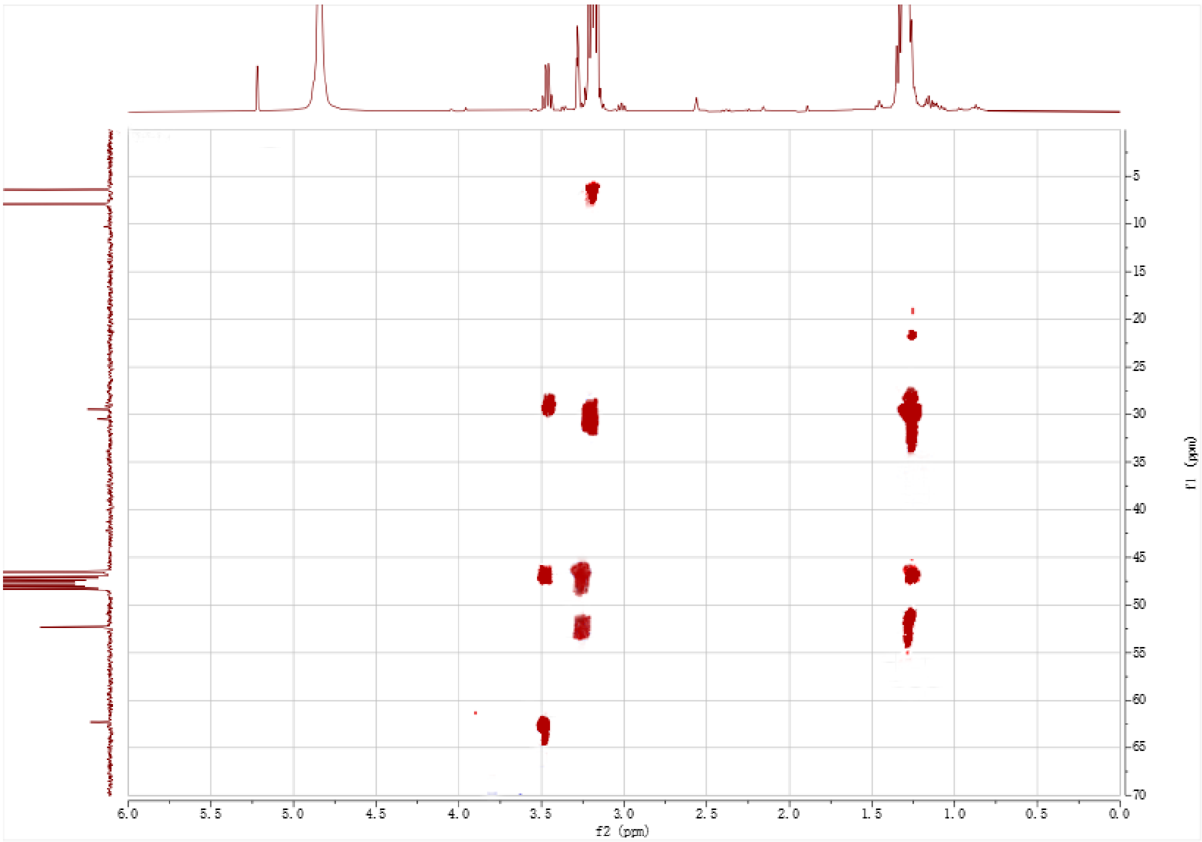

Figure S21. The NOESY spectrum of compound **3** in CD_3OD

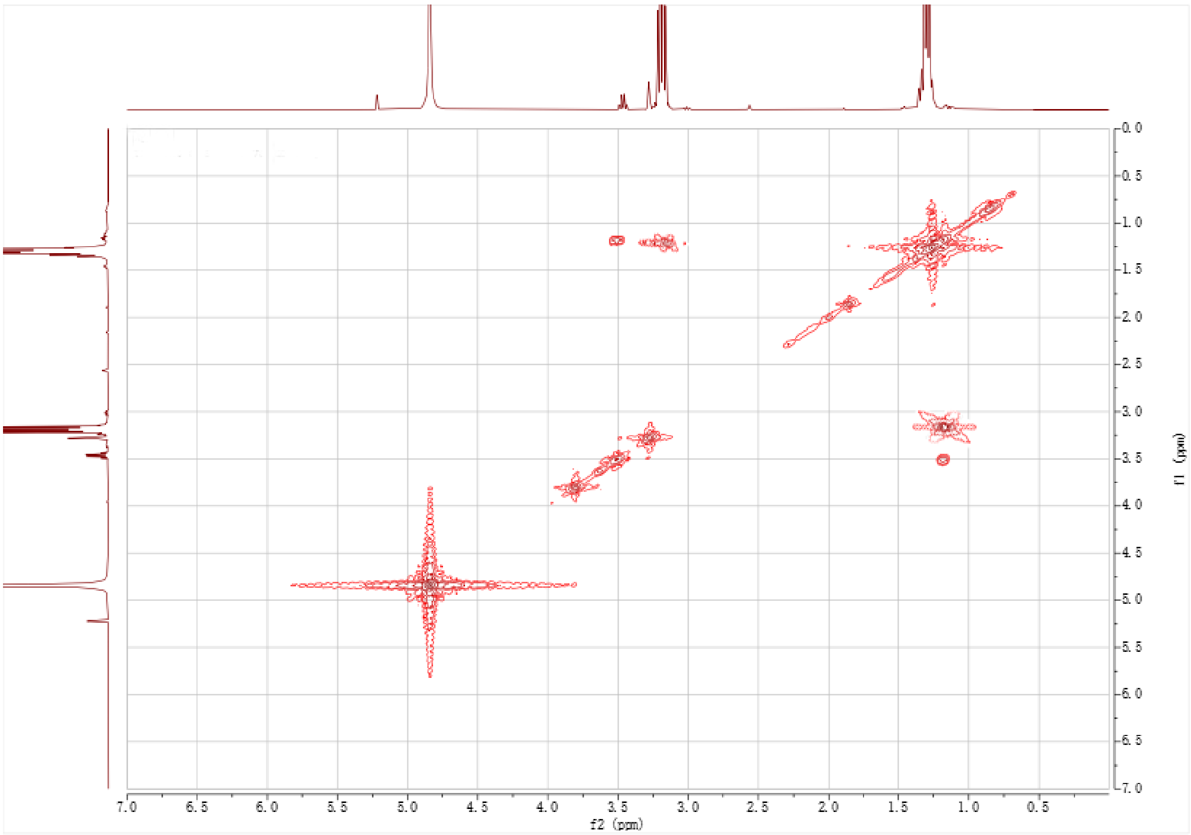

Figure S22. The HRESIMS spectrum of compound **3**

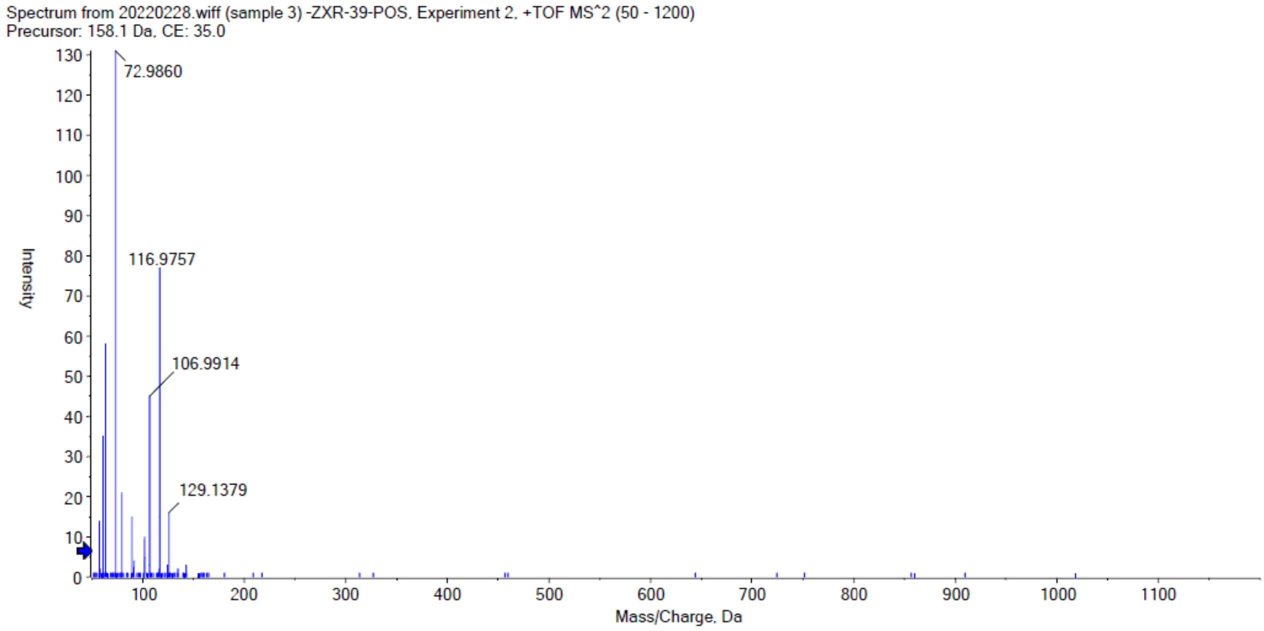

Figure S23. The ^{1}H NMR (400 MHz) spectrum of compound **4** in CD_3OD

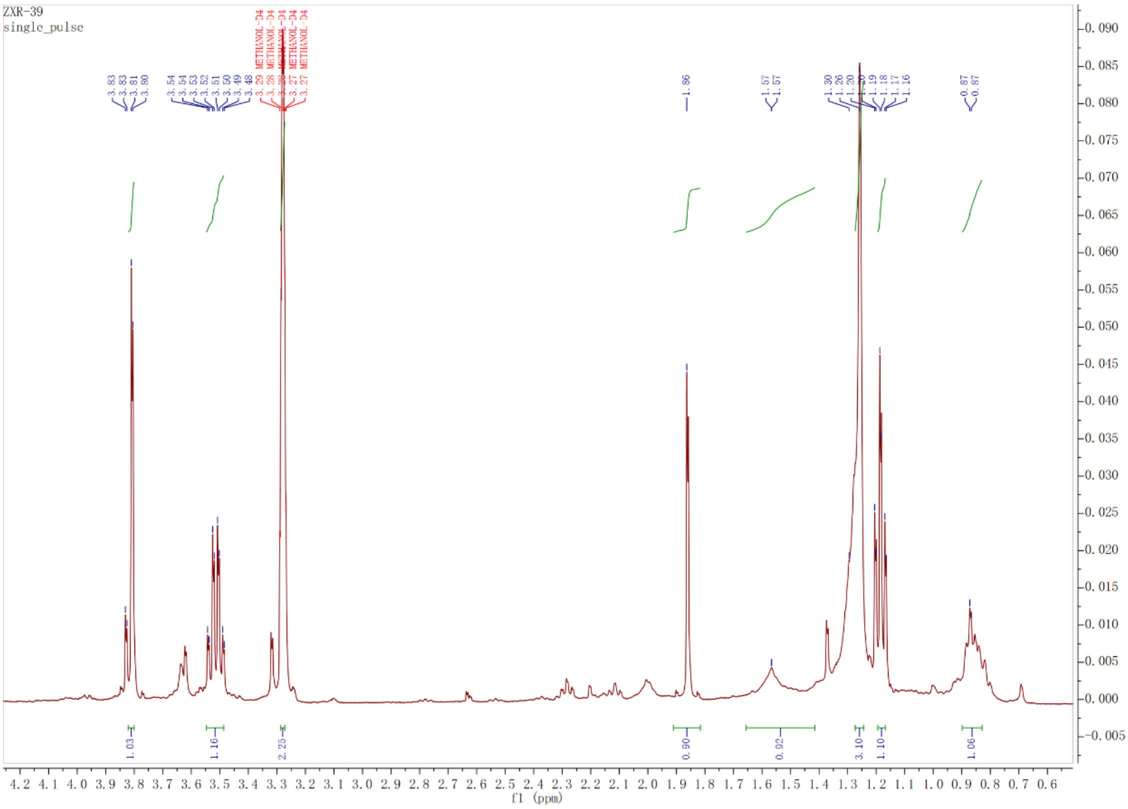

Figure S24. The ^{13}C NMR (100 MHz) spectrum of compound **4** in CD_3OD

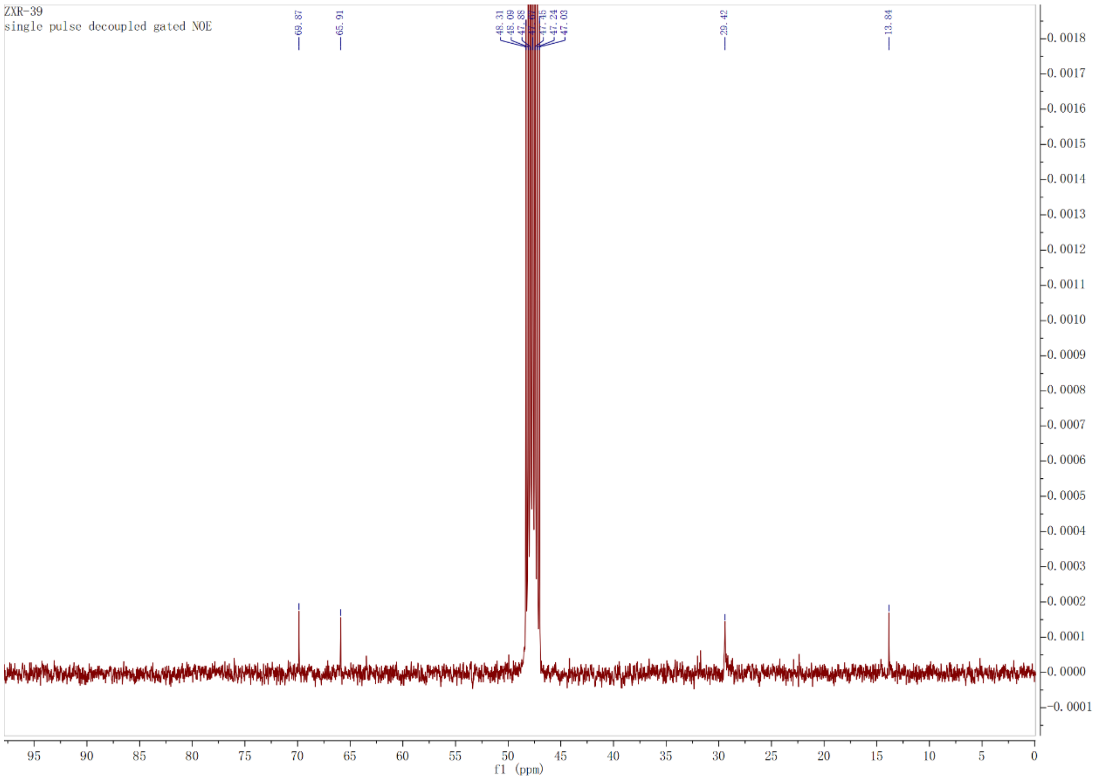

Figure 25. The HSQC spectrum of compound **4** in CD_3OD

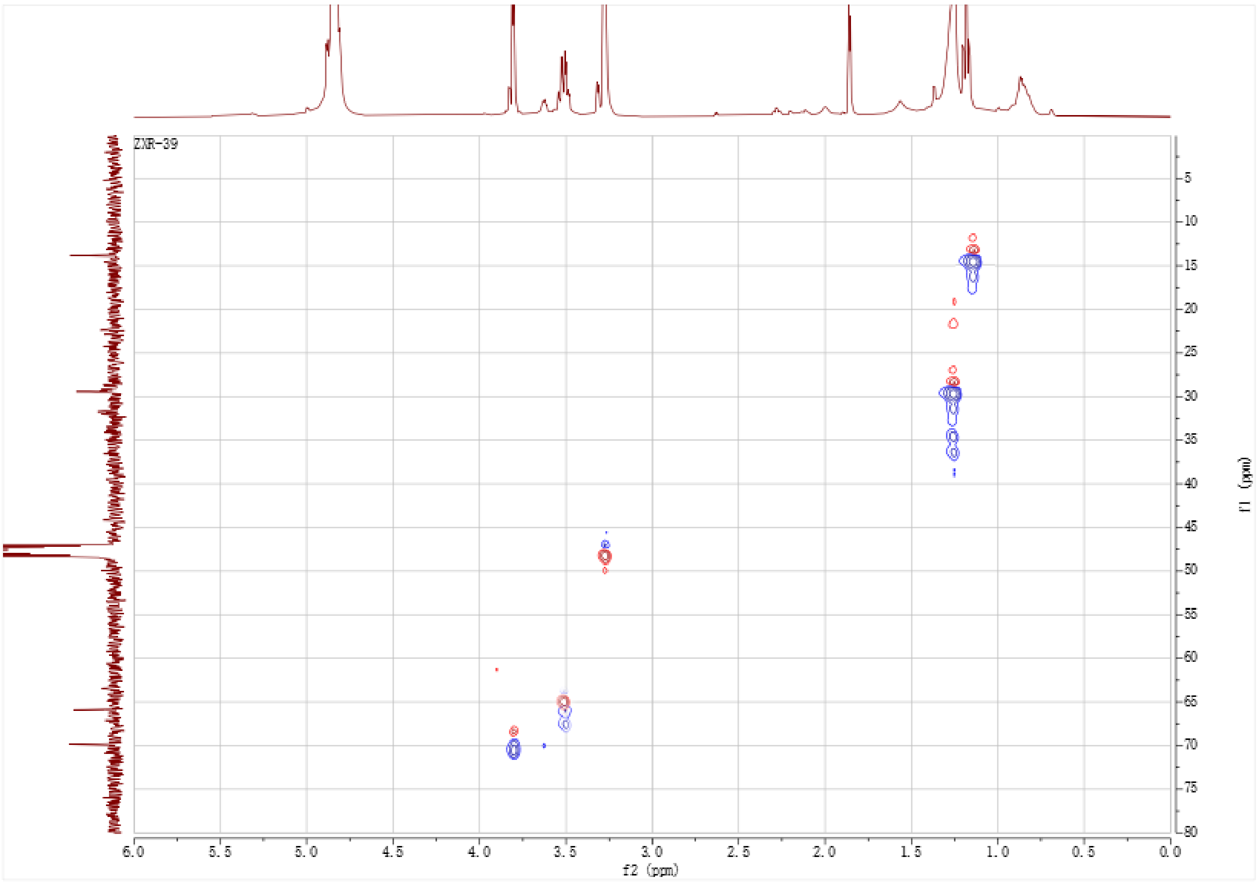

Figure 26. The HMBC spectrum of compound **4** in CD_3OD

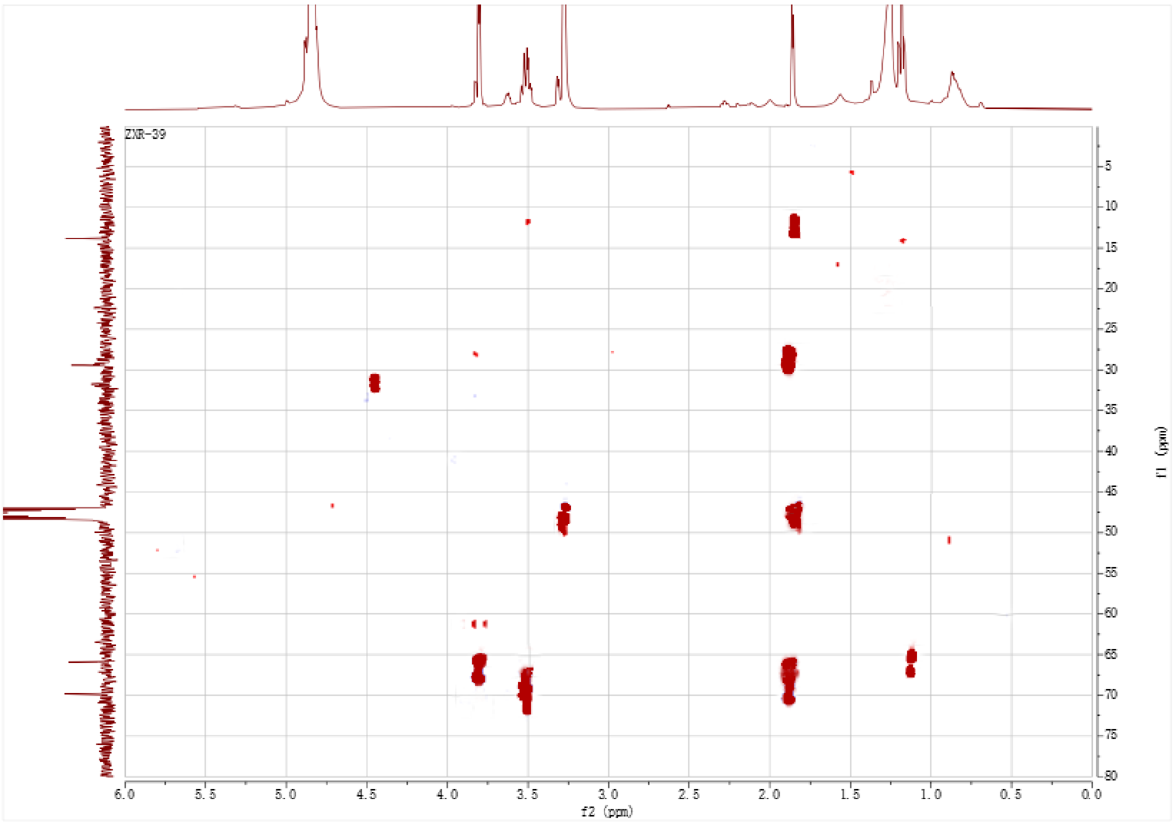

Figure S27. HRESIMS spectrum of compound **4**

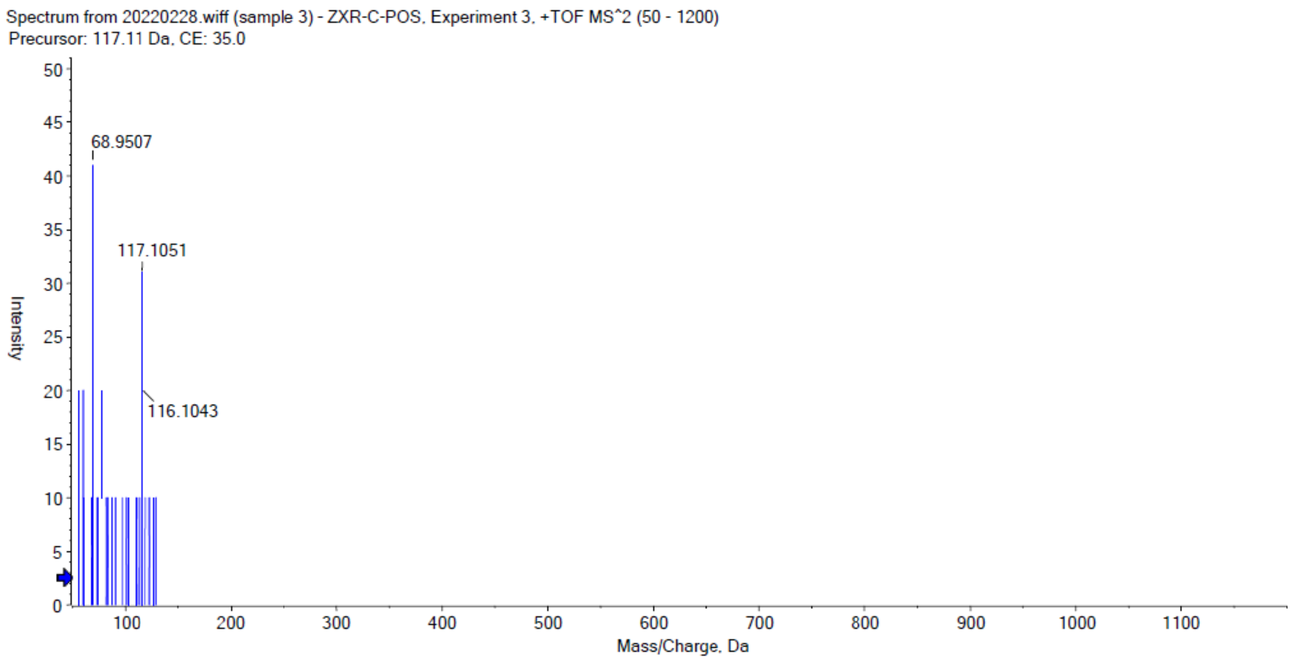

Figure S28. The ^{1}H NMR (400 MHz) spectrum of compound **5** in CD_3OD

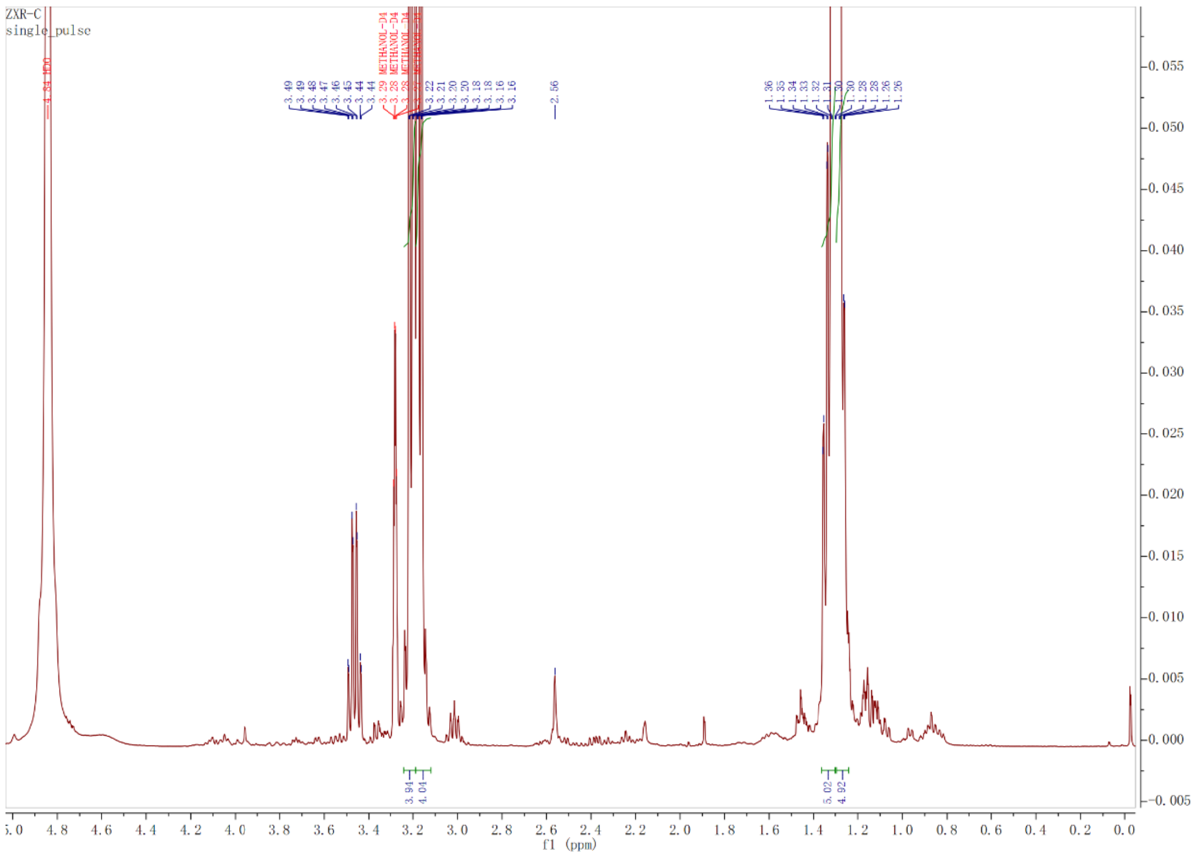

Figure S29. The ^{13}C NMR (100 MHz) spectrum of compound **5** in CD_3OD

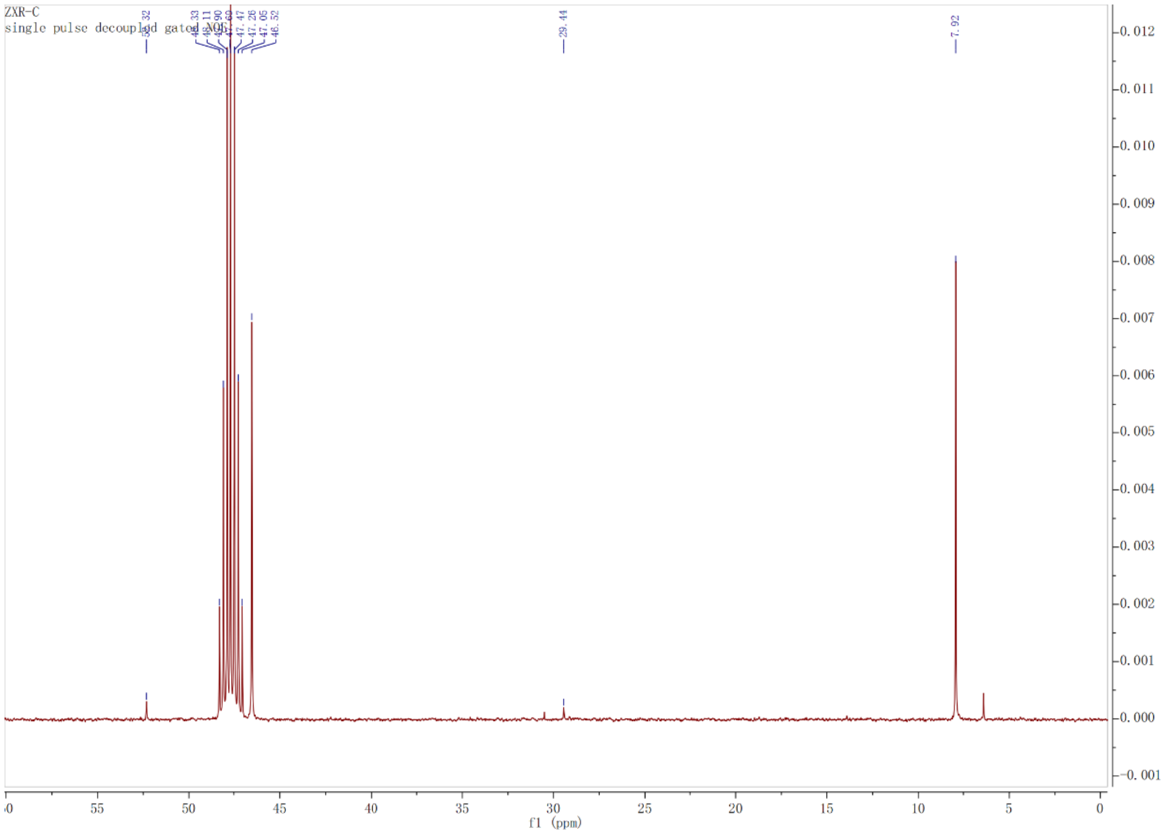

Figure S30. The HRESIMS spectrum of compound 5

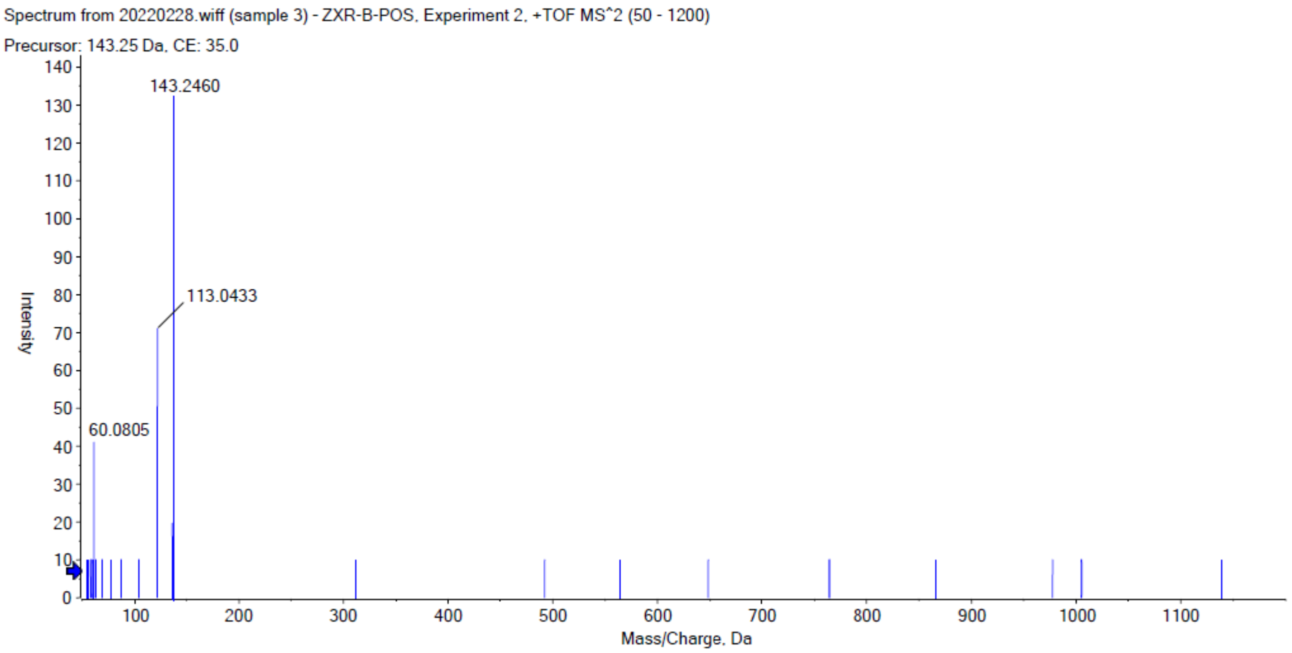

Figure S31. The ^{1}H NMR (400 MHz) spectrum of compound **6** in CD_3OD

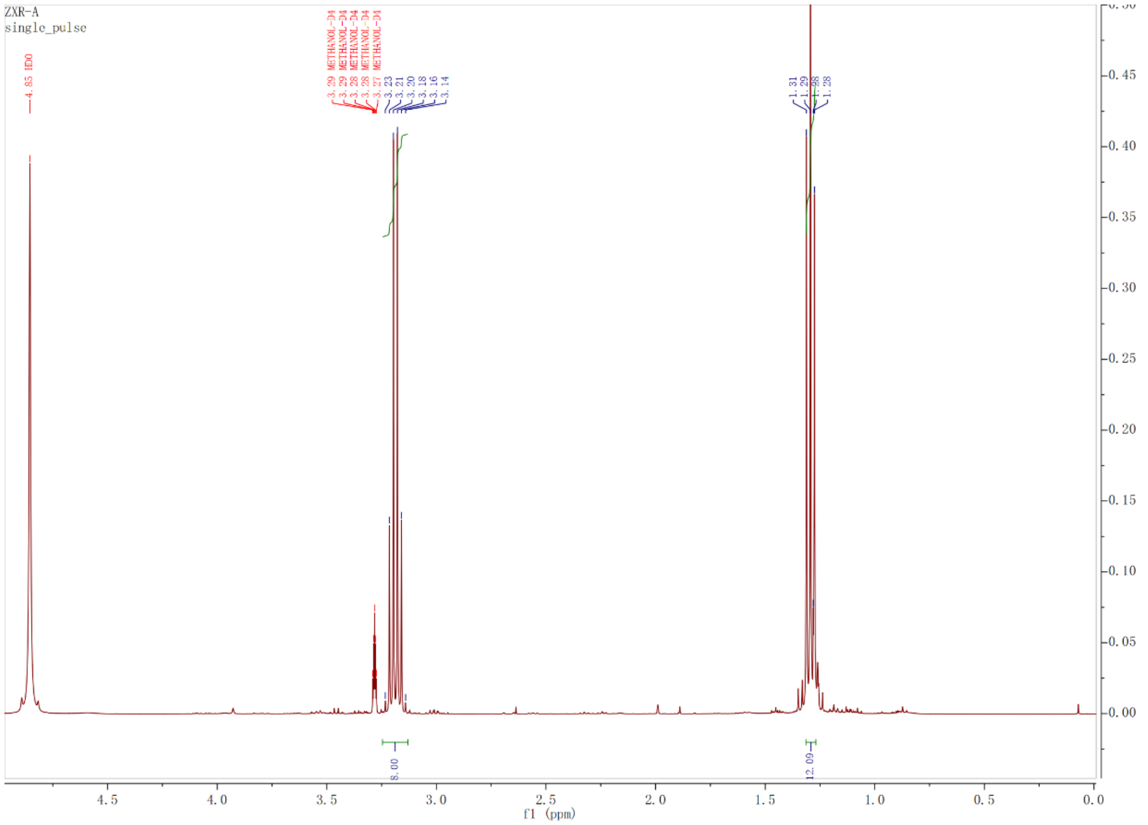

Figure S32. The ^{13}C NMR (100 MHz) spectrum of compound **6** in CD_3OD

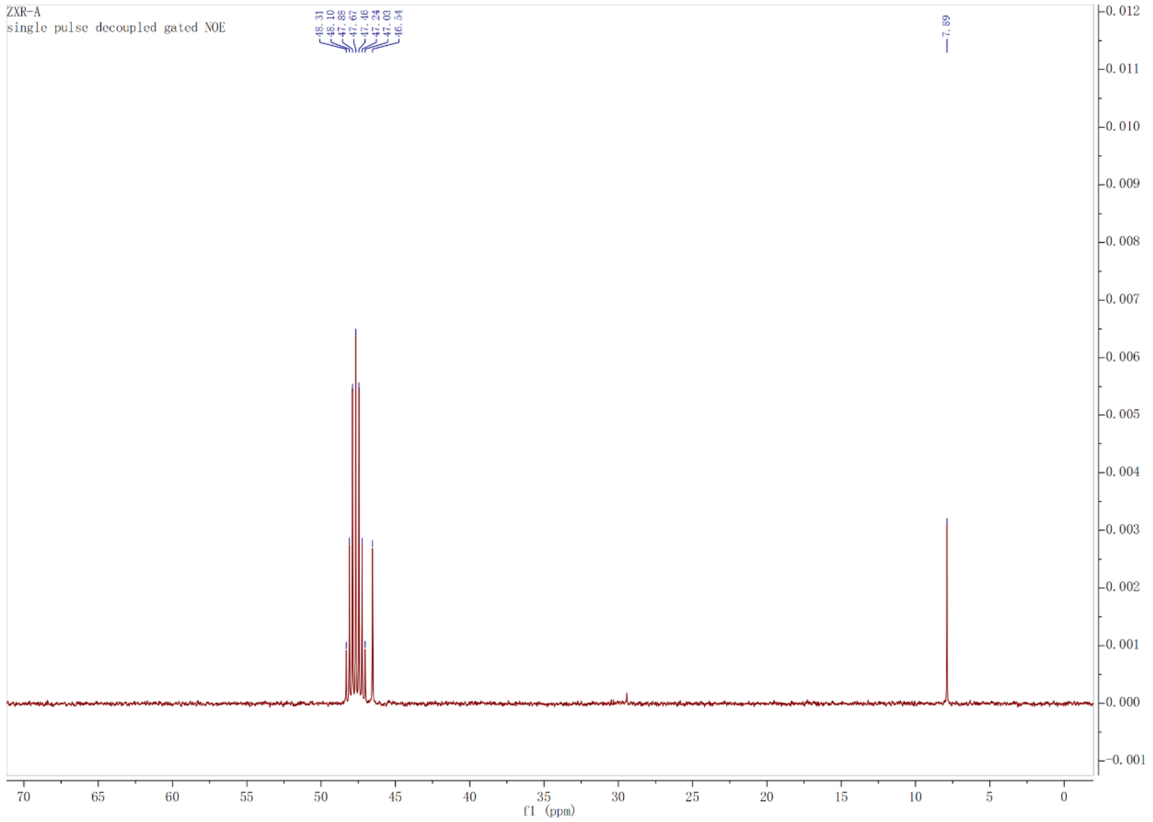

Figure S33. The HRESIMS spectrum of compound 6

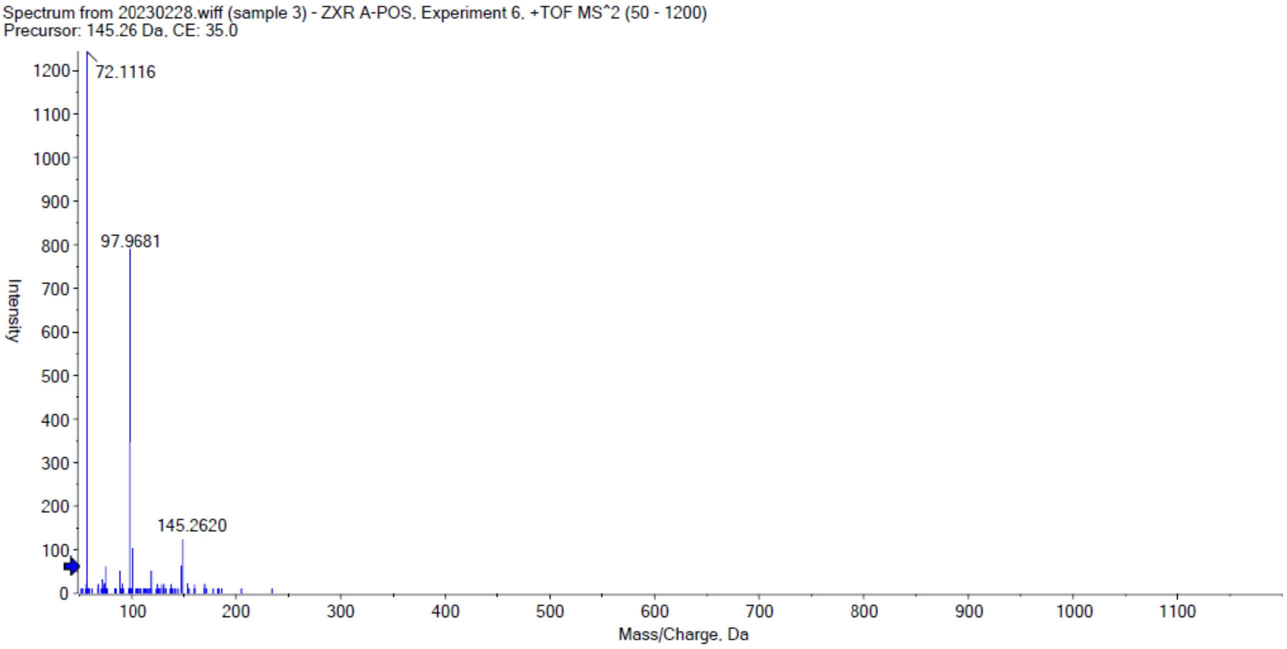

Supplement: Supplementary file 1 [file molecules-29-04446-s001.zip › molecules-3193573-supplementary.pdf]
